# Genetic Determinants of Endurance: A Narrative Review on Elite Athlete Status and Performance

**DOI:** 10.3390/ijms252313041

**Published:** 2024-12-04

**Authors:** Barkın Bıçakçı, Paweł Cięszczyk, Kinga Humińska-Lisowska

**Affiliations:** Faculty of Physical Education, Gdansk University of Physical Education and Sport, 80-336 Gdańsk, Poland; barkin.bicakci@awf.gda.pl (B.B.); pawel.cieszczyk@awf.gda.pl (P.C.)

**Keywords:** VO_2max_, exercise, polymorphism, physiology, heredity, multi-omics approach, cardiovascular fitness, energy metabolism, skeletal muscle, angiogenesis

## Abstract

This narrative review explores the relationship between genetics and elite endurance athletes, summarizes the current literature, highlights some novel findings, and provides a physiological basis for understanding the mechanistic effects of genetics in sport. Key genetic markers include *ACTN3* R577X (muscle fiber composition), *ACE* I/D (cardiovascular efficiency), and polymorphisms in *PPARA*, *VEGFA*, and *ADRB2*, influencing energy metabolism, angiogenesis, and cardiovascular function. This review underscores the benefits of a multi-omics approach to better understand the complex interactions between genetic polymorphisms and physiological traits. It also addresses long-standing issues such as small sample sizes in studies and the heterogeneity in heritability estimates influenced by factors like sex. Understanding the mechanistic relationship between genetics and endurance performance can lead to personalized training strategies, injury prevention, and improved health outcomes. Future studies should focus on standardized classification of sports, replication studies involving diverse populations, and establishing solid physiological associations between polymorphisms and endurance traits to advance the field of sports genetics.

## 1. Introduction

In sports science and especially sports genetics, elite athletes represent a critical population for study. These athletes push the boundaries of human physical performance, allowing researchers to explore fundamental questions such as the interplay between nurture and nature, the effects of specific training modalities, coaching strategies, and the very definition of talent. By studying elite athletes, researchers can explore the upper limits of human physiological capabilities and the genetic factors that contribute to exceptional performance. This knowledge not only advances the field of sports science but also has broader implications for public health, as cardiorespiratory fitness (CRF) is a key indicator of cardiovascular health and overall mortality risk. Despite the importance of defining elite athlete status, there is no universally established empirical definition [1]. However, the most commonly accepted criterion is competition at the highest national and international levels in a given sport [2,3,4]. In this review, we define elite athletes as individuals who have achieved top rankings in international competitions, such as world championships or the Olympic Games, and who have dedicated significant time and training to reach the pinnacle of their sport [2,3,4]. This definition includes athletes who consistently perform at a level significantly above the general athletic population, demonstrating exceptional physiological capacities and skill levels.

The lack of a universally accepted definition of ‘elite athlete’ presents challenges in research, particularly regarding the comparability of studies. Different studies may use different criteria to classify athletes as elite, such as performance metrics, competition levels, years of experience, or subjective assessments [5]. This variability can lead to inconsistencies in participant selection and categorization, making it difficult to compare results across studies or to perform meta-analyses [6]. For example, one study may define elite athletes based on national rankings, while another may require international competitive experience, leading to discrepancies that affect the generalizability of findings [6]. To address this issue, researchers need to clearly define their criteria for elite status and, when possible, use standardized definitions or classification systems to improve comparability between studies [1,7].

Establishing a physiological threshold for elite athlete status is very challenging due to the varying physiological demands of different sports [1,7]. Nonetheless, when compared with sedentary individuals or lower-level athletes in their respective sports, elite athletes consistently demonstrate superior physiological and performance capacities [7]. In particular, elite endurance athletes have significantly higher levels of CRF, often measured by maximal oxygen uptake (VO_2max_), than non-elite athletes and the general population [7,8]. Investigating the genetic factors that contribute to these superior CRF levels may help elucidate the biological pathways involved in aerobic performance and identify potential genetic markers associated with exceptional endurance performance.

Elite athletes can be categorized based on the physiological demands of their sport. Power and speed-oriented athletes specialize in activities requiring explosive strength and anaerobic capacity, such as powerlifting, 100 m sprints, discus throwing, 100 m swimming, and strongman events [3,9]. These sports mainly require bursts of maximal effort over a very short period of time [10].

In contrast, endurance athletes engage in sporting activities that require sustained power production, achieved through repeated isotonic contractions of large skeletal muscle groups over extended periods or distances. By efficiently using oxygen through aerobic metabolism, these muscle contractions enable the body to sustain prolonged physical activity [11]. Endurance sports include activities such as marathon running, cycling, triathlons, long-distance swimming, rowing, pentathlons, cross-country skiing, and ultramarathons [1,7,12,13,14,15]. The exceptional performance of elite endurance athletes suggests that genetic factors play a significant role in their ability to sustain high-intensity exercise over prolonged periods. Understanding these genetic factors, particularly those affecting CRF, is crucial for unravelling the biological basis of endurance performance.

For an elite endurance athlete, a high level of CRF is essential [16]. CRF is defined as the capacity of the cardiorespiratory system to deliver oxygen and substrates to muscles during exercise [8,17]. While a high CRF is a key indicator of athletic performance, it also plays a key role in general human health by serving as a protective factor against cardiovascular disease, a leading cause of all-cause mortality [17,18,19]. For endurance athletes, however, a high CRF is a requirement for competing at an elite level [7]. The interplay between genetic predisposition and environmental factors such as training and nutrition contributes to the development of high CRF levels in elite athletes. Investigating the genetic determinants of CRF may provide insight into individual variability in endurance performance and the potential for personalized training programs tailored to an athlete’s genetic profile.

Given its importance, the measurement and quantification of CRF is essential for monitoring both exercise performance and health outcomes. Maximal oxygen uptake (VO_2max_) is the gold standard for assessing CRF and endurance performance [8,20]. VO_2max_ is defined as the maximum rate at which an individual can consume oxygen during intense or maximal exercise [8]. It reflects a person’s aerobic fitness and is an important determinant of endurance capacity. VO_2max_ is typically measured in milliliters of oxygen consumed per kilogram of body weight per minute (mL/kg/min) [21]. A higher VO_2max_ indicates a greater ability of the heart and lungs to deliver oxygen to the working muscles and the muscles’ efficiency in using that oxygen to produce energy (ATP) through aerobic metabolism [8,22]. VO_2max_ can be assessed using incremental exercise tests on a treadmill or cycle ergometer, where the intensity is gradually increased until exhaustion [8]. For elite endurance athletes, VO_2max_ is used as both an indicator of endurance performance capacity and a developmental tool for optimizing training or exercise modalities [8,23]. Although contemporary sports science incorporates additional markers, such as blood lactate thresholds to supplement both research and performance enhancement [24], VO_2max_ remains central to endurance sports [25,26,27,28].

VO_2max_ is a complex trait influenced by both genetic and environmental factors, with limitations imposed by physiological factors such as cardiac output and pulmonary diffusing capacity [8,20,29]. Genetic factors account for approximately 44% to 68% of the interindividual variation in VO_2max_ and VO_2max_ response to training [20,29,30]. Thus, the role of genetics in the observed variation is undeniable. However, VO_2max_ is a polygenic trait influenced by multiple genes as well as single nucleotide polymorphisms (SNPs), including interactions between different SNPs (SNP–SNP interactions) [31]. SNPs are genetic variations that occur when a single nucleotide (adenine [A], thymine [T], cytosine [C], or guanine [G]) in the DNA sequence is altered [32]. If such a variation is present in at least 1% of the population, it is classified as a SNP [32,33]. SNPs are the most common type of genetic variation among people and form the basis of many genetic studies because they can influence how genes function and how individuals respond to environmental factors, including exercise.

Understanding these genetic and physiological factors may be important in developing personalized training interventions. The polygenic nature of VO_2max_ is closely linked to various physiological systems (e.g., cardiorespiratory and neuromuscular). By mapping an athlete’s SNP profile, researchers may gain insights into their current physiological status and their potential for adaptation through training. Although this field is still in its infancy and not yet widely used in sports practice, future advances could allow coaches and trainers to use genetic information to design personalized training programs tailored to the athlete’s genetic predispositions. For example, if genetic analysis indicates that an athlete has a predisposition for strong cardiorespiratory fitness but weaker muscular endurance, training could be adjusted to focus more on improving muscle fatigue resistance rather than just increasing VO_2max_. This personalized approach has the potential to make training strategies more efficient, potentially leading to faster improvements and optimized performance outcomes [34,35].

Gene–environment interactions also play a critical role in shaping VO_2max_, as environmental factors such as training intensity, altitude exposure, and nutritional status can modulate the expression of genetic potential. Understanding the complexity of these genetic influences and interactions is essential to fully elucidate the determinants of elite endurance performance.

Despite advances in sports genetics, the genetic determinants of elite endurance athlete status remain only partially understood, with inconsistent findings and a lack of clarity regarding the polygenic nature of endurance traits. This gap in knowledge is partly due to methodological challenges such as small sample sizes, population stratification, and the complex interplay of multiple genetic and environmental factors. Inconsistencies in findings across studies do highlight the need for more comprehensive research that integrates genetic data with physiological and environmental variables. Addressing these gaps is crucial for advancing our understanding of the genetic architecture underlying elite endurance performance and for translating this knowledge into practical applications in sports and health sciences.

Bray et al. [31] contributed one of the earliest compilations of sports-related genes and polymorphisms. In the seventh edition of the Human Gene Map for Performance and Health-Related Fitness Phenotypes, they added over 20 new genes to the map, bringing the total to 221 autosomal and X-linked genes, along with 18 mitochondrial markers. In later years, with the introduction and spread of new research strategies and methods, more focused and detailed genetic compilation studies in sports science have been published. One of the most up-to-date works is the review by Semenova et al. [27]. These authors examined literature on sports genetics and provided extensive detail on genes and polymorphisms that affect sports. According to Semenova et al. [36], as of May 2023, the total number of DNA polymorphisms associated with athletic performance is 128; of those 128 markers, 41 are endurance-related, 45 are power-related, and 42 are strength-related.

While the review by Semenova et al. [36] provides a comprehensive overview of genetic polymorphisms associated with athletic performance, our review aims to build on this foundation by focusing specifically on the integration of genetic findings with physiological data to understand the mechanistic effects of genetics in endurance sports. We concentrate on recent studies from the last five years that have examined not only known polymorphisms but also less-studied genes and new discoveries that have emerged since the publication of Semenova et al. [36]. In addition, we emphasize a bottom–up approach using multi-omics in the hypothesis generation phase by examining product–product interactions in candidate gene selection, which differs from traditional top–down methods. In this way, we aim to provide new insights into the polygenic complexity of endurance traits and highlight potential areas for future research.

This review aims to fill the aforementioned gaps in the literature by addressing the following research questions: (1) What are the key genetic factors influencing VO_2max_ and endurance performance in elite athletes, and how do these factors contribute to the polygenic complexity of endurance traits? (2) How do these genetic factors interact with physiological systems to influence endurance performance? (3) What are the methodological challenges in current genetic studies of endurance athletes or performance, particularly with regard to study design and sample size, and how can these challenges be overcome? (4) What future research directions are needed to advance our understanding of the genetic basis of endurance performance? By focusing on these questions, this review aims to synthesize current knowledge on the genetic factors influencing VO_2max_ and endurance performance by assessing and highlighting how these genes interact with physiological systems.

By examining the sports genetics literature relevant to elite athlete status, this review also aims to highlight the potential benefits of integrating genetic and physiological data to better understand the molecular mechanisms underlying endurance performance and development. Furthermore, by focusing attention on some of the less studied genes and recent discoveries, we aim to provide innovative directions for future research.

We hypothesize that integrating genetic findings with physiological data will improve our understanding of the complex interactions between genetic polymorphisms and physiological traits that influence endurance performance. In addition, we propose that addressing methodological challenges such as small sample sizes and population homogeneity will improve the reliability and replicability of genetic associations in endurance studies.

While the use of multi-omics approaches is not entirely novel in genetics research, we propose that applying multi-omics at the hypothesis generation stage represents an innovative approach in sports genetics. By adopting a bottom–up methodology, we suggest that integrating genomics, transcriptomics, proteomics, and metabolomics can help identify candidate genes based on physiological pathways and product–product interactions. This contrasts with traditional top–down approaches that start with known genes and look for associations. A better understanding of physiology, including how it is affected by epigenetic factors, might play a pivotal role in identifying which genes to examine. By using multi-omics data in this way, researchers can uncover new genetic factors that contribute to endurance performance, leading to a more comprehensive understanding of the underlying mechanisms. This approach could be achieved by examining product–product interactions in the candidate gene selection process, providing a novel perspective in the field.

We discuss the current understanding of the genetic basis of VO_2max_ and endurance performance, including key genes and genetic variants identified in recent studies. We examine how these genetic factors interact with physiological systems to influence endurance performance. We also critically review the methodological challenges in genetic studies of endurance, emphasizing the need for robust study designs and larger, more diverse cohorts. Finally, we identify gaps in current research and suggest future directions, emphasizing the importance of integrating genetic findings with physiological and training data to improve our understanding of elite endurance performance. By addressing these questions and highlighting the current limitations of sports genetics research, this review aims to provide a comprehensive understanding of the genetic factors that influence elite endurance performance. This knowledge has the potential to inform the development of personalized training strategies, improve talent identification processes, and contribute to the broader field of precision medicine.

## 2. Genes and Polymorphisms and Their Association with Endurance Performance

### 2.1. Materials and Methods

A comprehensive literature search was conducted across four databases—PubMed, Science Direct, Cochrane, and Google Scholar—from 14 April 2024 to 6 May 2024. The aim was to identify polymorphisms and their combinations that require further investigation in the context of endurance performance. Polymorphisms were considered for investigation if: (1) they had shown inconsistent or conflicting results in previous studies regarding their association with endurance performance or VO_2max_; (2) they were identified in recent studies but lacked extensive research or replication; (3) they were located in genes with known physiological relevance to endurance performance but had not been extensively studied in elite endurance athletes; (4) they were novel or understudied polymorphisms emerging from recent genetic studies, such as genome-wide association studies (GWAS), suggesting potential associations with endurance performance.

Studies were also considered for inclusion if they met additional criteria: published in the last 5 years (from 1 January 2019 to 30 April 2024), compared elite endurance athletes with sedentary controls or other athlete groups if they had a dedicated endurance subject group, examined mixed-sport athletes, and studies with only athlete groups. Articles written in English only were considered. Exceptions were made if the articles had abstracts in another language, but the full text was written in English, as long as the content met our inclusion criteria. In such cases, abstracts were translated using well-established online translation tools, such as Deepl, to ensure accuracy and consistency in the selection process. Studies focusing on genes and their effect on VO_2max_ and VO_2max_ trainability were sought using Boolean operators AND/OR, and the following keywords to guide this search: “single nucleotide polymorphisms”, “SNPs”, “cardiorespiratory fitness”, “cardiovascular fitness”, “polymorphism”, “endurance”, “elite athletes”, “VO_2max_, “association”, “sports”, and “maximal oxygen uptake.” Titles and abstracts were initially screened to select relevant studies, and full texts were reviewed when abstracts lacked sufficient information. Two authors independently reviewed the search results to identify studies for inclusion. In cases where discrepancies arose, a third reviewer was engaged to resolve disagreements and make the final decision, ensuring a more reliable and unbiased selection process.

To critically evaluate genetic studies on VO_2max_ and endurance performance, we implemented a standardized study quality scoring system adapted from Hennis et al. [37] and based on the work of Clark and Baudouin [38]. This scoring system, outlined in Table 1, provides a comprehensive assessment of the methodological rigor and validity of the included studies.

### 2.2. Results

We focused specifically on the most recent original publications from the past five years (from January 2019 to April 2024), as shown in Table 2. These studies underwent a thorough check for scientific quality, with the results summarized in Table 3.

Recent studies have highlighted the polygenic nature of endurance performance and emphasized a shift toward studying multiple genes rather than focusing on single-gene effects [39,40,41]. Table 2 summarizes the characteristics of this work.

**Table 2:** Characteristics of the included studies.

Study	Gene(s)	Polymorphism(s)	Participants	Endurance Related Associations
de Albuquerque-Neto et al., 2024 [42]	*ACTN3* *ACE* *BDKRB2* *AGT*	R577X (rs1815739) I/D (rs4343) −9/+9 (rs5810761) M268T(rs.699)	123 Brazilian swimmers (*n* = 19 elite, 104 sub-elite) Brazilian controls (*n* = 718)	XX genotype of the R577X was associated with endurance performance
Akazawa et al., 2022 [43]	*ACTN3*	R577X (rs1815739)	Japanese elite athletes (*n* = 906) divided into subgroups Japanese controls (*n* = 649)	No association in endurance athletes were found
Ben-Zaken et al., 2020 [39]	*IL-6* *IGFBP3*	174G/C (rs1800795) A-202C (rs2854744)	Israeli elite athletes (*n* = 113 endurance, *n* = 110 sprint/power) Israeli controls (*n* = 64)	*IL-6* C and *IGFBP3* C alleles were significantly greater among long-distance swimmers compared with long-distance runners
Bosnyák et al., 2020 [44]	*HIF1A* *GNB3*	Pro582Ser (rs11549465) C825T (rs5443)	Caucasian elite athletes (*n* = 110 endurance, *n* = 38 sprint/power) Caucasian controls (*n* = 90)	No association
Bulgay et al., 2024 [45]	*AMPD1*	C34T (rs17602729)	Caucasian elite athletes (*n* = 29 endurance, *n* = 31 sprint/power) Caucasian controls (*n* = 20)	No association in endurance athletes
Bulgay et al., 2023 [46]	*VDR*	Fok1 (rs2228570)	Caucasian elite athletes (*n* = 29 endurance, *n* = 31sprint/power) Caucasian controls (*n* = 20)	No association
Chiu et al., 2019 [47]	*ACE*	I/D (rs1799752)	Ultra-marathon runners (*n* = 24)	D allele was associated with better ultramarathon performance when not adjusted for body fat
El Ouali et al., 2024 [48]	*CD36*	G/A (rs1761667)	Moroccan elite endurance athletes (*n* = 43) Moroccan controls (*n* = 28)	No significant difference between athletes and controls
Guilherme et al., 2021 [49]	*MCT1*	A1470T (rs1049434)	Elite Brazilian and European endurance athletes (*n* = 318, *n* = 315) Brazilian and European controls (*n* = 890, *n* = 552)	T allele was associated with endurance performance
Hall et al., 2023 [40]	*PPARGC1A*	Gly482Ser (rs8192678)	British elite endurance athletes (*n* = 288) British controls (*n* = 368)	482Ser allele carriers were faster compared with Gly/Gly homozygotes
Jacob et al., 2021 [50]	*ACE* *ACTN3* *ADRB1* *PPARGC1A*	I/D (rs4343) R577X (rs1815739) Arg389Gly (rs1801253) Gly482Ser (rs8192678)	Elite players in the Australian Football League (*n* = 46)	*ADRB1* Arg389Gly CC and *PPARGC1A* Gly482Ser GG genotypes were associated with better endurance performance
Kanope et al., 2022 [51]	*LIN28A*	A/G (rs6598964)	Brazilian soccer players (*n* = 38 professional, *n* = 189 U20/17/15)	Predicted VO_2max_ was associated with the *LIN28A* A/A genotype
Kazanci et al., 2023 [52]	*IL-6* *PPARA*	-597A/G (rs1800795) G/C (rs4253778)	Elite cross-country skiing athletes (*n* = 30)	IL-6 GC and *PPARA* GG genotypes are associated with endurance performance
Paulo Guilherme et al., 2019 [53]	*FTO*	T/A (rs9939609)	Brazilian and Russian athletes (*n* = 677 Brazilian, *n* = 920 Russian) Brazilian and Russian Controls (*n* = 652 Brazilian, *n* = 754 Russian)	A/A genotype of *FTO* T/A polymorphism is underrepresented in long-distance athletes and combat sports athletes of lighter weight categories
Piscina-Viúdez et al., 2021 [54]	*MCT1*	A1470T (rs1049434)	Spanish endurance athletes (*n* = 88) Spanish controls (*n* = 107)	Overrepresentation of TT genotype among athlete groups
Sierra et al., 2019 [55]	*AGT* *ACE* *BDKRB2*	Met235Thr (rs699) I/D (rs4343) −9/+9 (rs5810761)	Brazilian marathon runners (*n* = 81)	*ACE* II, *AGT* Met/Met, and *BDKRB* −9/+9 genotypes were associated with higher myocardial and skeletal muscle inflammation
Végh et al., 2022 [56]	*ACTN3* *ACE* *HIF1A* *PPARA*	R577X (rs1815739) I/D (rs4646994) Pro582Ser (rs11549465), G/C (rs4253778)	Slovak elite athletes (*n* = 23 endurance running, *n* = 41 football) Slovak controls (*n* = 54)	*ACE* II, *HIF1A* Pro/Ser, and C allele of the *PPARA* G/C were advantageous regarding super-compensation
Vostrikova et al., 2022 [57]	*ACE* *BDKRB2* *NOS3* *PPARGC1A*	I/D (rs4646994) −9/+9 (rs5810761) G894T (rs1799983) Gly482Ser (rs8192678)	Highly qualified martial artists (*n* = 50) Low qualification martial artists (50)	Gly/Gly genotype of Gly482Ser and I/D genotype of the *ACE* I/D polymorphisms were more frequent in highly qualified martial artists
Wojciechowicz et al., 2021 [24]	*KIF6* *APOE*	Trp719Arg (rs20455) Cys130Arg (rs429358) Arg176Cys (rs7412)	Elite Polish athletes (*n* = 104 endurance, *n* = 100 sprint/power) Polish controls (*n* = 164)	No association
Yang et al., 2023 [41]	*ACE* *ACTN3*	I/D (rs4343) R577X (rs1815739)	Chinese youth athletes (*n* = 73 elite, *n* = 69 sub-elite) Chinese youth controls (*n* = 107)	XX genotype of the R577X was associated with endurance performance

*ACE*—Angiotensin-Converting Enzyme, *ACTN3*—Alpha-actinin-3, *ADRB1*—Adrenoceptor Beta 1, *AGT*—Angiotensinogen, *AMPD1*—Adenosine Monophosphate-1, *APOE*—Apolipoprotein E, *BDKRB2*—Bradykinin B2 receptor, *CD36*—Cluster of Differentiation 36, *FTO*—Fat Mass and Obesity-associated Gene, *GNB3*—Guanine Nucleotide-Binding Protein Subunit beta-3, *HIF1A*—Hypoxia Inducible Factor 1 Subunit Alpha, *IGFBP3*—Insulin-like Growth Factor Binding Protein 3, *IL-6*—Interleukin 6, *KIF6*—Kinesin Family Member 6, *LIN28A*—Lig-28 Homolog A, *MCT1*—Monocarboxylate Transporter 1, *NOS3*—Nitric Oxide Synthase 3, *PPARA*—Peroxisome Proliferator-Activated Receptor Alpha, *PPARGC1A*—Peroxisome Proliferator-Activated Receptor Gamma Coactivator 1-Alpha, *VDR*—Vitamin D Receptor.

There is growing recognition of the importance of a multi-omics approach [46,58] that integrates genomics with other “omics” technologies to better understand the complex interactions between polymorphisms and their effects on physiology. This holistic approach promises to unravel the intricate genetic underpinnings of endurance performance. Better understanding of physiology with all its aspects, including how it is affected by epigenetic factors, might play a pivotal role in identifying which genes to be examined. While it should be noted that the usage of multi-omics in the methodology of genetics research is not novel, the use of multi-omics in the hypothesis stage of study development might help refine the approach taken (e.g., bottom–up versus top–down). This could be achieved by examining product–product interactions in the candidate gene selection process.

An example of this approach is a study by Sierra et al. [55], which focused on the *ACE* I/D, *AGT* Met235Thr, and *BDKRB2* −9/+9 polymorphisms and found that the *ACE* II, *AGT* Met235Met, and *BDKRB2* −9/+9 genotypes were associated with increased muscle damage and inflammatory markers after an ultramarathon. Although the study had a relatively small sample size of 81 Brazilian male runners and was limited to a specific demographic, its findings have major implications for both medical and sports science. The association of these genotypes with increased muscle damage suggests that certain genetic profiles may predispose athletes to higher levels of physiological stress during extreme endurance events. This could have implications for personalized training and recovery protocols, as athletes with these genotypes may benefit from targeted interventions to mitigate muscle damage and inflammation. Furthermore, understanding these associations enhances our knowledge of the molecular mechanisms underlying exercise-induced muscle damage, potentially leading to novel therapeutic strategies.

However, even with a multigenic approach, the inconclusive nature of the literature persists. One of the most likely reasons for the status quo is that while a physiology-based approach might strengthen theory and prove useful in discovering mechanistic pathways, it does not address the limitations of genetic association studies.

Genetic association studies examine specific genes that are thought to influence sport-related traits. In these studies, polymorphisms within the selected genes are analyzed to determine any allelic effects on the trait of interest [59]. The frequency of these polymorphisms is then compared between different populations, typically athletes and non-athletes. If polymorphism frequency is significantly greater among athletes versus a control group, one can infer a link to athletic status and/or performance [36,60]. Such studies are often supplemented with physiological data, such as the athlete performance metrics, to strengthen the findings. On the other hand, Genome-Wide Association Studies (GWAS) take a hypothesis-free approach, analyzing genetic markers across the genome to identify potential genes and polymorphisms associated with specific traits. This method uses microarrays to measure hundreds of thousands or millions of SNPs simultaneously [60]. Despite its strengths, GWAS is limited by its lack of direct physiological or genetic mechanisms, but its contribution to sports science is undeniable [12].

While these studies provide valuable insights, they also face significant methodological challenges, particularly with respect to sample size [36,59,60]. By their nature, elite athletes represent an incredibly small percentage of the general population, and performance-related polymorphisms are inherently rare [60]. Studies with limited participant numbers are more prone to false positive errors, which can lead to over- or underestimation of effect sizes [36]. These errors, combined with the impact of small sample sizes on statistical power, make study replication and accurate conclusions difficult to obtain [36,60].

A study by Guilherme et al. [49] involved one of the largest sample sizes among the studies reviewed and examined the Glu490Asp polymorphism (rs1049434, A1470T) of the *MCT1* gene in a diverse cohort of Brazilians (318 endurance athletes and 890 controls) and Europeans (315 endurance athletes and 552 controls) for endurance athlete status. *MCT1* encodes a transporter for lactate, and the A1470T polymorphism is thought to affect the transporter’s function, thus affecting endurance performance by influencing energy metabolism. Athletes’ metrics were evaluated by examining blood lactate and VO_2max_. The study found that carriers of the T allele were more likely to be endurance athletes, and those with the TT genotype had lower blood lactate levels during and after maximal exercise when compared with TT homozygotes. Ethnic differences in genotype distribution also emerged, whereby the AA genotype was underrepresented in individuals of African descent (roughly 35% of the total Brazilian cohort). These findings suggest that the T allele may confer an advantage in lactate clearance and utilization during intense exercise, potentially enhancing endurance performance. The observed ethnic difference highlights the importance of considering population genetics in sports genomics research while underscoring the need for recruiting diverse populations in genetics research to ensure findings are broadly applicable.

These studies, despite their limitations, illustrate the benefits of a multigenic and multiethnic approach to genetic research. Ideally, association studies should include thousands of elite athletes and controls to achieve sufficient statistical power. Even higher sample sizes are required for GWAS [59]. However, this is often impossible due to the limited number of elite athletes available for research, especially when considering the need for subgroup allocations (by sport), which further reduces group size and statistical power [12].

Several well-researched polymorphisms, such as *ACTN3* R5XX7 and *ACE* I/D, have yielded somewhat associations with elite athlete status. However, the multiethnic composition of populations, such as those in Brazil, adds complexity to genetic studies, as genetic diversity can significantly influence results [43,49,53,55]. To date, most research has focused on Caucasian populations [60]. However, allele frequencies vary between populations [45,61], and different populations, such as Ethiopian runners with high-altitude adaptations, have unique genetic profiles [59]. Brazilian, Asian (predominantly Chinese and Japanese), and Slavic cohorts are among many diverse populations that exhibit different genetic profiles compared with each other [4]. This genetic diversity also contributes to the low generalizability and replicability of genetic studies across populations. Contemporary data suggest that ethnicity may play a major role in the effect of certain polymorphisms on elite athlete status and/or endurance performance [39,41,51,52]. For example, the prevalence of the *ACE* I allele, which has been associated with endurance performance, varies among different ethnic groups, potentially influencing the distribution of endurance-related traits across populations. Understanding these variations is fundamental to interpreting genetic association studies and developing population-specific strategies for talent identification and training programs. Therefore, it is crucial for future studies to include diverse populations and consider ethnic-specific allele frequencies when analyzing genetic associations.

Replication studies are essential to confirm associations between polymorphisms and performance-related traits [12,36]. To establish a robust association, the genetic variant of interest must be consistently replicated in a similar population [36]. Such replication allows for the compilation of higher-level evidence-based studies, including meta-analyses.

Another critical consideration in genetic studies is the accurate grouping of athletes. The challenge of accurately grouping athletes and conducting position-specific analysis in team sports, especially those that require both endurance and strength, such as football or martial arts, can further complicate research findings [43,50,57]. It is crucial to consider the specific demands of each sport and position when conducting genetic studies, but doing so inevitably reduces sample size and statistical power.

In a study of elite Slovak endurance athletes, the I allele and II genotype of the *ACE* I/D and the C allele of the *PPARA* rs8192678 polymorphism were shown to have a favorable effect on supercompensation, a key factor in recovery and performance enhancement [56]. In addition, the I allele of *ACE* I/D and the G allele of *PPARA* rs8192678 were more frequently associated with endurance athletes compared with strength-oriented athletes. This study also examined other polymorphisms, such as *ACTN3* R577X and *HIF1A* rs11549465, but found that the *ACTN3* R577X did not significantly impact supercompensation [56]. These findings suggest that a polygenic approach offers greater potential for understanding the genetic contributions to endurance performance. In other words, multiple favorable alleles may enhance physiological adaptations to training, as a direct consequence of supercompensation, thereby improving endurance performance. This underscores the potential of using genetic information to predict training responses and tailor individualized training programs. Still, the lack of association with *ACTN3* R577X suggests that not all commonly studied polymorphisms are valid genetic markers in different populations or performance measures, emphasizing the complexity of genetic influences on endurance.

In contrast, Chiu et al. [47] found that the D allele of the *ACE* I/D was associated with better ultramarathon performance in a primarily Taiwanese cohort (*n* = 24, I/I = 7, I/D = 14, D/D = 3). However, no statistically significant differences were observed after adjustment for BMI. In addition, physiological parameters were examined (e.g., lipid profiles), but no significant differences between the different *ACE* polymorphisms were reported. Despite the small sample size, the authors suggested that the different physiological demands of ultramarathons, when compared with traditional marathons, may explain these findings. It is attractive to hypothesize that the influence of certain polymorphisms, such as *ACE* I/D, may be context-dependent, varying with the specific endurance discipline or environmental conditions. Larger cohort studies are needed to confirm these associations and to explore how different endurance events may interact with genetic factors to influence performance.

As discussed earlier, sports can generally be categorized into endurance sports, which are predominantly aerobic in nature, and sprint/power sports, which are largely anaerobic. However, many sports, such as basketball, mixed martial arts, or tennis (mixed sports), have both endurance and power demands [60]. Therefore, it is important to accurately define the physiological demands of a given sport and classify it accordingly. This is challenging in the case of mixed sports, given that any misclassification can lead to false positives, or more likely, no genetic association. Moreover, even sports that fall into the same category, such as powerlifting and 100 m swimming, which can be categorized as anaerobic sports, may have different determinants of elite performance [60]. A further consideration is position-specific demands in team sports. For example, rugby players can be categorized into two different subgroups, forwards and backs. Forwards engage in more high-intensity static activities, while backs engage in more high-intensity running [62]. Therefore, when conducting studies on athletes from different sports, the subgrouping procedure should be scrutinized. Failure to account for these nuances may obscure true genetic associations or lead to misleading conclusions. Recognizing the specific physical and metabolic demands of each sport, or positional demands within a sport, allows for a more precise examination of how genetic factors contribute to performance and may increase the ability to detect significant associations.

The literature on genetic associations with endurance performance remains inconclusive, as evidenced by conflicting results for polymorphisms such as *ACE* I/D [50]. As discussed before, *ACE* I/D and *BDKRB2* −9/+9 have been associated with sports performance. In contrast, Jacob et al. [50] found no association between any *ACE* I/D genotype and performance metrics in elite Australian football athletes (*n* = 46). While this study did not have a control group and only examined genes associated with increased performance, the very small sample size may have contributed to false negative results. Without adequate statistical power, studies like this may fail to detect true associations, leading to inconsistent results when compiling literature in this field. Reported effects (or lack thereof) are also compounded by a lack of an appropriate control group.

Some studies have reported no significant associations for other polymorphisms such as *GNB3* and *CD36.* The *GNB3* C/T polymorphism, in which the T allele is associated with enhanced G-protein activity, has garnered interest because of its role in various signalling pathways (e.g., blood pressure response, heart rate modulation). However, despite a slightly higher prevalence of the T allele in endurance athletes, no significant association with endurance performance was found [44]. Another study by Sawczuk et al. [63] examined Polish elite athletes (*n* = 223, 123 endurance athletes and 100 strength/power athletes) and Caucasian controls (*n* = 354). Sawczuk et al. [63] found no associations between the groups across the *GNB3* C/T genotypes. Furthermore, İpekoğlu et al. [64] conducted an analysis of seven articles, including those by Sawczuk et al. [63] and Eynon et al. [65], encompassing a total of 805 endurance athletes and 2146 controls. This comprehensive analysis found no significant associations between *GNB3* C/T and endurance athlete status. These findings suggest that the *GNB3* C/T polymorphism may not play a significant role in determining endurance performance or that its effect size may be too small to detect without larger sample sizes. Additionally, these results highlight the need for replication studies and meta-analyses to validate initial positive findings to ensure that reported associations are robust and not due to chance or study-specific factors.

Similarly, CD36, a key protein involved in fat metabolism, has been studied for its potential impact on endurance performance. The G/A polymorphism (rs1761667) of the *CD36* gene is thought to affect the expression of *CD36* and consequently endurance performance. In a study that examined 43 elite Moroccan athletes (19 cyclists and 24 field hockey players), the G allele was overrepresented, while the AA genotype was absent in the cyclists, suggesting a possible negative effect of the A allele in endurance sports. This negative effect may be due to reduced *CD36* expression associated with the A allele, resulting in decreased fatty acid uptake and utilization, which is essential for endurance activities [48]. However, when athletes were compared with controls, no significant differences in genotype distribution were found, suggesting that *CD36* alone may not be a determinant of athlete status [48]. One possibility is that the *CD36* effect on athletic status is limited or modulated by other genetic and environmental factors. This highlights the complex nature of endurance performance, where multiple genes and their interactions contribute to the phenotype. The lack of any significant findings in these studies might again be due to small samples, which may cause false negatives. On the other hand, the *CD36* G/A polymorphism is an interesting case, as this polymorphism is relatively novel, and there are insufficient published data to develop a cohesive athlete profile. This single study, albeit limited by its participant numbers, brings up an interesting area of research.

*CD36* plays a vital role in the regulation of whole-body long-chain fatty acid (LCFA) uptake and oxidation. Exercise has been shown to increase *CD36* mRNA and protein expression in skeletal muscle [66]. Physically active individuals may exhibit higher *CD36* mRNA expression as compared with sedentary individuals due to regular exercise-induced upregulation of *CD36*. This upregulation enhances LCFA (long-chain fatty acid) uptake and oxidation, providing a more efficient energy source during prolonged exercise [66]. In endurance-trained athletes, a correlation was found between *CD36* and peak fat oxidation, suggesting that increased *CD36* levels contribute to improved fat utilization during endurance exercise [66]. This increased capacity for fat oxidation is beneficial for endurance performance by conserving glycogen stores and delaying fatigue [67]. Furthermore, prolonged endurance exercise training has been shown to increase CD36 protein levels along with an increased ability to oxidize fat [48]. These physiological findings suggest that genetic variations affecting *CD36* expression or function may have significant effects on endurance performance. Further research with larger cohorts is warranted to investigate the potential role of *CD36* polymorphisms in endurance performance, which may reveal novel targets for performance enhancement and/or metabolic interventions.

Along with CD36, other transport proteins might be an interesting area for researchers (see Table 2). The results of these studies indicate that further investigation is necessary, employing multiple perspectives based on molecular and physiological understanding and utilizing large, diverse sample sizes, to fully elucidate the genetic basis of endurance performance. While some polymorphisms have consistently been associated with endurance traits, others remain controversial. These conflicting results may arise from limitations in sample size or variations in study quality, as summarized in Table 3, which assesses the quality of the studies listed in Table 2. Consequently, there is a clear need for replication studies and further research to validate these findings.

A critical analysis of the methodological design of selected studies, as presented in Table 3, reveals considerable variability in research quality. Items were scored “1” if the criterion was met (“Yes”) and “0” if not (“No”). For case-control studies, the maximum possible score was 10 points, while for cohort studies, it was either 8 or 9 points, depending on the applicability of certain criteria.

**Table 3:** Quality analysis of the included studies.

Study ID	Control Group	HW Equilibrium	Case Group/Whole Group	Primer	Reproducibility of Genotyping	Blinding	Power Calculation	Statistics	Corrected Statistics	Independent Replication	Total
Case-Control											
de Albuquerque-Neto et al., (2024) [42]	1	1	1	1	1	0	0	1	1	0	7/10
Akazawa et al., (2022) [43]	1	1	1	0	1	0	0	1	1	0	6/10
Ben-Zaken et al., (2020) [39]	0	1	1	1	1	0	0	1	1	0	6/10
Bosnyak et al., (2020) [44]	0	1	1	0	1	0	0	1	0	0	4/10
Bulgay C Et al., (2024) [45]	0	1	1	1	1	0	1	0	1	0	6/10
Bulgay C, et al., (2023) [46]	0	1	1	0	1	0	0	1	1	0	5/10
El Ouali et al., (2024) [48]	0	1	1	1	1	0	0	1	1	0	6/10
Guilherme et al., (2021) [49]	1	1	1	0	1	0	0	1	1	1	7/10
Paulo Guilherme et al., (2019) [53]	1	1	1	1	1	0	0	1	1	0	7/10
Hall et al., (2023) [40]	1	1	1	0	1	0	0	1	1	0	6/10
Piscina-Viúdez et al., (2021) [54]	1	1	1	0	1	0	0	1	1	0	6/10
Végh et al., (2022) [56]	0	0	1	1	1	0	0	1	1	0	5/10
Wojciechowicz et al., (2021) [58]	0	1	1	1	1	0	1	1	1	0	7/10
Yang et al., (2023) [41]	0	1	1	1	1	0	0	1	1	0	6/10
Cohort											
Chiu et al., (2019) [46]	-	-	1	1	1	0	0	1	1	0	5/8
Jacob et al., (2021) [50]	-	0	1	0	0	0	0	1	0	0	2/9
Kanpoe, et al., (2022) [51]	-	1	1	1	1	0	0	1	1	0	6/9
Kazancı et al., (2023) [52]	-	0	0	0	1	0	1	0	0	0	2/9
Sierra et al., (2019) [55]	-	1	1	1	1	0	0	1	1	0	6/9
Vostrikova et al., (2022) [57]	-	-	1	1	1	0	0	1	1	0	5/8

Most of the case-control studies scored between 5 and 7 out of a possible 10 points, indicating moderate quality. Studies such as de Albuquerque-Neto et al. [42], Guilherme et al. [49], and Wojciechowicz et al. [58] achieved higher scores (7/10), reflecting stronger methodological rigor. These studies included the use of control groups, assessment of the Hardy–Weinberg equilibrium, detailed genotyping methods (e.g., provision of primer sequences), evidence of genotyping reproducibility, and appropriate statistical analyses with corrections for multiple testing. Conversely, some studies had lower scores due to a lack of control groups, lack of power calculations, or insufficient statistical corrections for multiple testing. For example, Bosnyák et al. [43] scored 4/10 using these criteria.

Among the cohort studies, scores ranged from 2 to 6 out of a possible 8 or 9 points, indicating variability in quality. Studies by Kanope et al. [51] and Sierra et al. [55] scored 6/9, demonstrating moderate quality by meeting criteria such as assessment of the Hardy–Weinberg equilibrium, adequate description of the study population, provision of primer information, reproducibility of genotyping, appropriate statistical analyses, and application of corrected statistics. In contrast, studies by Jacob et al. [50] and Kazanci et al. [52] scored only 2/9, due to a failure to meet many of these conditions.

This variability in study quality may contribute to inconsistent findings observed in the literature. That is, studies with higher methodological rigor are more likely to produce reliable and replicable results, whereas studies with methodological limitations may produce results that are less reliable and biased. This highlights the importance of adhering to strict methodological standards in genetic research to increase the validity of any conclusions drawn.

As research in the field of sports genetics advances, our understanding of the genetic complexity underlying endurance performance also evolves. Each new study that examines different polymorphisms adds to the growing body of knowledge, drawing clearer connections between genetic variants and tangible changes in athletic performance.

Collectively, these findings highlight the need for rigorous methodological approaches in future research, including larger sample sizes, standardized protocols, and consideration of the complex interactions between multiple genes and environmental factors. By addressing these methodological challenges, researchers can better isolate the effects of individual genes and understand their contributions to endurance performance.

## 3. Mechanisms of Genetic Influence on CRF and VO_2max_

As previously discussed, genetics plays a critical role in determining VO_2max_ and, consequently, elite athlete status. Nonetheless, the specific mechanisms through which genes influence VO_2max_ are complex and multifaceted. To better understand how genes affect VO_2max,_ it is essential to examine the physiological pathways and limitations associated with VO_2max_ as outlined by Basset and Howley [8]. This section focuses on how genetic factors influence key physiological determinants of VO_2max_, specifically the ability of the lungs to transfer oxygen to the blood (pulmonary diffusing capacity), the ability of the blood to carry oxygen (oxygen-carrying capacity), the amount of blood the heart pumps per minute (cardiac output), and how skeletal muscles function during exercise.

### 3.1. Pulmonary Diffusing and Oxygen-Carrying Capacity

Oxidative phosphorylation, the process of generating ATP from oxygen and nutrients, is critical for meeting the energy demands of endurance activities [68]. This process takes place in the mitochondria, where ATP is produced and subsequently used to power various bodily functions [68]. Given the central role of oxygen in ATP production, understanding the mechanisms of oxygen transport from the atmosphere to the mitochondria is critical. The body’s ability to diffuse and transport oxygen is a key physiological determinant of VO_2max_, which in turn is a primary indicator of endurance performance [69,70]. For endurance athletes, optimizing these oxygen transport mechanisms may be the key to improving performance. However, there has been little discussion of how genetic factors specifically influence each step of this oxygen transport pathway.

The pathway of oxygen from the lung to the mitochondria is well described in the literature [71]. Briefly, oxygen is first inhaled into the lungs, then diffuses across the alveolar capillary membrane into the pulmonary capillary blood, where it binds to hemoglobin. Oxygen is then transported throughout the body via the cardiovascular system and finally diffuses from the red blood cells to the mitochondria, where it is used for energy production [71]. Genetic polymorphisms can affect each step of the oxygen transport pathway. For example, certain genes can affect lung function, the ability of the blood to carry oxygen, or how effectively muscles use oxygen. The efficiency of this process, known as pulmonary diffusion capacity—defined as the ability of the lungs to transfer gases from the alveoli to the blood—is a key factor in determining VO_2max_. Pulmonary diffusing capacity has been shown to be influenced by both genetic and environmental factors [72]. Recent studies, such as that of Dridi et al. [73], indicate that intense endurance training (IET) significantly enhances pulmonary diffusing capacity, nitric oxide diffusing capacity, pulmonary capillary blood volume, and alveolar–capillary membrane function compared with moderate training (MET), highlighting the importance of exercise intensity in improving pulmonary vascular function and potentially VO_2max_. Comparative studies have shown that endurance athletes generally have higher pulmonary diffusing capacity versus sedentary individuals, likely due to both genetic predisposition and training-induced adaptations [74]. One gene that may influence pulmonary function is *NOS3* (Nitric Oxide Synthase 3), which is involved in the production of nitric oxide, a molecule that helps regulate blood vessel dilation [75]. Specific polymorphisms in *NOS3*, such as the Glu298Asp (rs1799983), can affect the production of nitric oxide, potentially influencing lung function and oxygen exchange [76]. However, more research is needed to fully understand their impact on elite endurance performance.

Oxygen-carrying capacity is another critical component of VO_2max_ and is primarily determined by the level of hemoglobin in the blood. Hemoglobin, a protein with an iron-containing heme group, significantly increases the blood’s ability to carry oxygen [77]. Higher hemoglobin levels may improve oxygen delivery to working muscles, thereby enhancing endurance performance.

Genetic factors influencing hemoglobin levels may, therefore, play a crucial role in an athlete’s endurance capacity. For example, the *EPO* (Erythropoietin) gene regulates the production of erythropoietin, a hormone that stimulates red blood cell (RBC) production. Polymorphisms in the *EPO* gene can lead to differences in hemoglobin levels between individuals [78,79,80].

Erythropoiesis is regulated by hypoxia-inducible factors (HIFs), which respond to oxygen levels in the blood. Exposure to high altitudes induces hypoxia, leading to increased EPO production and subsequently higher hemoglobin levels [81]. These findings suggest that genetic variations affecting the *HIF* or *EPO* pathways may also influence an individual’s ability to adapt to hypoxic conditions, potentially enhancing endurance performance. For example, polymorphisms in the *HIF1A* gene, such as Pro582Ser (rs11549465), have been studied for their potential effects on aerobic performance, although results to date are inconclusive [82,83].

When EPO binds to EPOR, it induces JAK2 (Janus kinase 2), which is a tyrosine kinase, to change its structure. This initiates a series of cascade reactions as multiple phosphorylation (addition of a phosphoryl molecule) events occur, and these phosphorylated sites act as docks for signaling molecules such as STAT5 (Signal Transducer and Activator of Transcription 5), which are transcription factors. STAT5 acts on many genes within the cell and, in turn, stimulates erythropoiesis. Genetic mutations affecting any component of this pathway could potentially enhance or inhibit red blood cell production, thereby affecting oxygen-carrying capacity.

A notable example is the G6002A mutation in the erythropoietin receptor *(EPOR)* gene, associated with erythrocytosis, a condition characterized by increased RBC production [84]. Percy et al. [85] found that this mutation introduces a stop codon, leading to the loss of phosphatase binding sites and impairing negative feedback mechanisms [85]. Consequently, the JAK2-STAT5 pathway remains activated for extended periods, resulting in elevated RBC production, higher hemoglobin levels, and increased oxygen-carrying capacity. This genetic alteration has been linked to the exceptional endurance performance of some elite athletes, like Eero Antero Mantyranta, suggesting a direct genetic influence on VO_2max_ and endurance capacity. Nevertheless, such mutations are rare and can also pose a health risk due to an excessive number of RBCs in the blood [84,85].

While they confer advantages in oxygen transport, they may also confer health risks associated with excessive erythrocytosis, such as an increased risk of thromboembolic and atherosclerotic disease due to chronically elevated EPO signalling and hyperviscosity of the blood [86]. This suggests that although the G6002A mutation is beneficial for endurance performance, its potential health detriments may explain its minimal presence in the general athletic population.

In summary, genetic factors influencing pulmonary diffusing capacity and oxygen-carrying capacity are crucial determinants of VO_2max_. While training can enhance these capacities, genetic predispositions may set the upper limits of an individual’s potential for endurance performance. Further research is needed to identify specific genetic polymorphisms that significantly impact these physiological processes in athletes.

### 3.2. Cardiac Output

Cardiac output (Q), which represents the amount of blood pumped by the heart per minute, is a key determinant of VO_2max_ [87]. Cardiac output is the product of heart rate (HR) and stroke volume (SV), and it is estimated that 70–85% of the limitation of VO_2max_ is due to maximal cardiac output [8,88]. Simply put, the more blood the heart can pump, the more oxygen that can be delivered to the working muscles. Given the importance of Q, understanding how genetic factors influence its regulation is essential for understanding endurance performance varies among individuals.

Stroke volume is determined by preload, afterload, and myocardial contractility, each of which can be influenced by genetic predisposition [89]. For example, genes that regulate myocardial wall thickness and venous/arterial compliance, such as those involved in the renin-angiotensin system (RAS), play an important role in determining stroke volume [90]. Prolonged endurance exercise may induce physiological changes in these factors, leading to exercise-induced physiological cardiac hypertrophy, commonly referred to as the “athlete’s heart” [91]. This hypertrophy is associated with increased stroke volume and cardiac output, thereby improving VO_2max_. However, there has been little discussion of the specific genetic variants that contribute to these cardiac adaptations in endurance athletes.

From a genetic perspective, the *ACE* gene, which encodes angiotensin-converting enzyme, has been extensively studied for its role in cardiovascular adaptations to endurance exercise. A common polymorphism in the *ACE* gene is the *ACE* insertion (I)/deletion (D) polymorphism, which has been associated with sports performance [92,93,94,95,96]. The I allele (insertion variant) has been linked to superior endurance performance, possibly due to lower *ACE* activity, which may lead to improved blood vessel dilation (vasodilation), reduced blood pressure, and decreased resistance against which the heart must pump, thereby improving cardiac efficiency [97]. Conversely, the D allele (deletion variant) and DD genotype are associated with higher *ACE* activity and have been linked to greater cardiac hypertrophy (or enlargement of the heart muscle) and power-oriented activities [93,98,99,100]. A likely explanation is that the D allele increases *ACE* activity, leading to increased levels of angiotensin II, which promotes cardiac muscle growth and vascular remodeling. This means that genetic variations in the *ACE* gene can influence how the heart adapts to endurance training, affecting an athlete’s potential for training-related performance gains.

Although the exact mechanism by which the *ACE* I/D polymorphism affects cardiac adaptations is not fully understood, the renin-angiotensin system (RAS) and associated microRNAs are thought to play a central role by regulating expression throughout the molecular pathways of cardiac hypertrophy [101,102]. Further study of these molecular pathways may provide a deeper understanding of how genetic variations influence cardiac adaptations in athletes.

Angiotensin II (Ang-2) acts as a potent vasoconstrictor and regulator of fluid balance, blood pressure, and myocardial growth within the RAS system. Increased production of Ang-2, facilitated by the ACE enzyme and the D allele, supports blood flow redistribution during intense exercise but, if unregulated, can also promote cardiac hypertrophy and maladaptive remodeling through pathways involving angiotensin II [103,104]. These genetic influences underscore the importance of considering the *ACE* I/D polymorphism when assessing athletic potential for elite endurance performance. However, the relationship between *ACE* genotype and endurance performance remains controversial, with some studies having reported conflicting findings [47,50,56,105]. These inconsistencies may be due to interactions with other genetic factors, environmental influences, or differences in study design and population genetics.

In addition to *ACE*, other genes such as *ADRB1* (Adrenergic Receptor Beta-1) and *ADRB2* (Adrenergic Receptor Beta-2) have been studied for their influence on heart rate and cardiac output during exercise. Polymorphisms in these genes may affect the sympathetic regulation of heart rate and contractility, further influencing endurance performance.

Understanding the genetic influences on cardiac output can provide insights into individual variability in VO_2max_ and endurance capacity. While training can induce significant cardiovascular adaptations, genetic predispositions may ultimately determine the extent of these adaptations and set limits to an individual’s potential.

### 3.3. Skeletal Muscles

Muscle fiber composition is a crucial factor in determining endurance performance. Skeletal muscle can be predominantly classified into two types: slow-twitch (type I) and fast-twitch (type II) fibers [106]. The distinction between these fiber types is based on their rate of contraction [107]. Type I fibers are characterized by slower contraction speeds and higher oxidative capacity, making them more efficient for sustained, aerobic activities. They are more resistant to fatigue and are ideal for endurance sports such as marathon running. In contrast, type II fibers contract more rapidly and rely more on glycolytic pathways, making them suitable for short, high-intensity efforts [107].

Type I and II muscle fibers produce energy in different ways. Type I muscle fibers, which predominate in endurance athletes, generate energy primarily through oxidative phosphorylation, giving them a higher oxidative capacity [108]. This higher oxidative capacity is supported by a greater abundance of mitochondria, capillaries, and myoglobin—key elements that facilitate oxygen transport and utilization within the muscle fiber [106,107,108]. In contrast, type II muscle fibers rely more on glycolysis (anaerobic respiration) for energy production, making them better suited for short, intense bursts of activity rather than sustained endurance efforts [107]. The predominance of type I fibers in endurance athletes is well documented, with studies showing that these athletes typically have a higher proportion of type I fibers compared with those involved in power sports such as wrestling, powerlifting, and sprinting [108].

Given the significant role of muscle fiber composition in endurance performance, understanding the genetic factors that influence muscle fiber type is essential. From a genetic perspective, the *PPARGC1A* gene plays a multifaceted role in athletic performance [40,109]. *PPARGC1A* encodes a transcriptional coactivator called PGC1α (Peroxisome Proliferator-Activated Receptor γ Coactivator 1α) [110], which regulates multiple genes that play via actions related to mitochondrial biogenesis, glucose and fatty acid oxidation, and fiber-type determination [109]. Overexpression of *PPARGC1A* has been associated with the conversion of type II muscle fibers to type I fibers, a process driven by increased activity of PGC1α [109]. This suggests that *PPARGC1A* may influence muscle fiber composition and trainability to selected exercises, thereby affecting endurance performance. However, the exact mechanisms by which specific genetic variants in *PPARGC1A* influence fiber-type transformation in humans remain unclear.

A specific single nucleotide polymorphism (SNP) in the *PPARGC1A* gene, known as Gly482Ser (G/A), has been associated with variations in athletic performance [40,109,110]. This polymorphism causes changes in the expression or activity of PGC1α. Interestingly, both the Gly and Ser alleles appear to influence athletic performance, although in different contexts [40,109]. A meta-analysis undertaken by Chen et al. [109] examined 10 studies and found out that the Gly allele was overrepresented in both endurance and power athletes, suggesting a general advantage in athletic performance. In contrast, Hall et al. [40] showed that, in Caucasian long-distance runners, Ser allele carriers were faster than Gly/Gly homozygotes. These conflicting results may be due to the influence of other genetic factors or gene–environment interactions that modulate the effect of the *PPARGC1A* polymorphism. It is possible that, due to the central role of PGC1α, any variation in *PPARGC1A* could have a cascading effect on mitochondrial function, energy metabolism, and muscle fiber composition, leading to different phenotypic outcomes. Further research is needed to elucidate the precise mechanisms involved and to determine whether these polymorphisms can be used as predictive markers of endurance performance or potential.

The complex role of *PPARGC1A* in muscle fiber composition highlights the importance of taking a molecular approach to fully understand the genetic influences on VO_2max_ and endurance performance. As discussed previously, VO_2max_ is a polygenic trait influenced by numerous genes that regulate key physiological systems such as the cardiovascular and respiratory systems [36]. These systems have complex physiologies, each influenced by a variety of genes that interact within and between molecular pathways.

**Figure 1:**
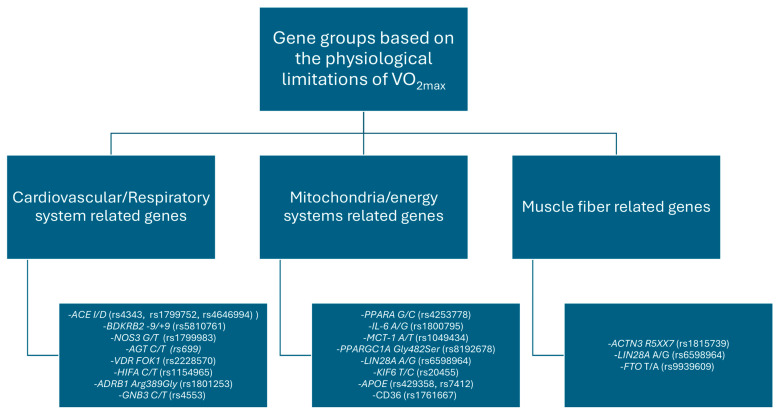
Gene groupings. *ACE*—Angiotensin-Converting Enzyme, *ACTN3*—Alpha-actinin-3, *ADRB1*—Adrenoceptor Beta 1, *AGT*—Angiotensinogen, *AMPD1*—Adenosine Monophosphate-1, *APOE*—Apolipoprotein E, *BDKRB2*—Bradykinin Receptor B2, *CD36*—Cluster of Differentiation 36, *FTO*—Fat Mass and Obesity-associated Gene, *GNB3*—Guanine Nucleotide-Binding Protein Subunit beta-3, *HIF1A*—Hypoxia Inducible Factor 1 Subunit Alpha, *IGFBP3*—Insulin-like Growth Factor Binding Protein 3, *IL-6*—Interleukin 6, *KIF6*—Kinesin Family Member 6, *LIN28A*—Lig-28 Homolog A, *MCT1*—Monocarboxylate Transporter 1, *NOS3*—Nitric Oxide Synthase 3, *PPARA*—Peroxisome Proliferator-Activated Receptor Alpha, *PPARGC1A*—Peroxisome Proliferator-Activated Receptor Gamma Coactivator 1-Alpha, *VDR*—Vitamin D Receptor.

To further illustrate the polygenic nature of endurance performance, we have grouped genes according to their primary physiological effects on endurance performance, focusing on their impact on the physiological limitations of VO_2max_. This categorization is illustrated in Figure 1.

Figure 1 categorizes key genes based on possible physiological targets, illustrating how genetic polymorphisms can influence the physiological limitations of VO_2max_. The cardiovascular/respiratory system genes influence oxygen delivery and cardiovascular efficiency, mitochondrial/energy metabolism genes influence cellular energy production, and muscle fiber composition genes determine muscle functionality. This categorization provides a framework to understand the multifactorial genetic contributions to endurance performance.

To highlight the diversity of genetic factors involved, we discuss several genes below, including some not included in Table 2 of our literature review, such as *VEGF* and *UCP3*, to demonstrate the need for more diversified research in this area.

#### 3.3.1. Alpha-Actinin-3 Gene (*ACTN3*)

As underlined above, genetic factors play a role in determining an individual’s muscle fiber composition. One such gene that has been extensively studied in relation to skeletal muscle function and endurance performance is the alpha-actinin-3 (*ACTN3)*. *ACTN3* encodes a protein called α-actinin-3, which plays a key role in force generation and explosiveness. This protein is mainly expressed in myocytes, especially in fast-twitch muscle fibers [46,111]. A polymorphism (rs1815739) in the *ACTN3* gene (exon 16) results in a nonsense mutation (R577X) whereby a cytosine (C) is replaced by a thymine (T) at nucleotide position 1747. This mutation introduces a premature stop codon, resulting in the absence of functional α-actinin-3 in individuals homozygous for the X allele (XX genotype) [112].

Interestingly, the presence of the X allele of the *ACTN3* polymorphism, which results in the absence of α-actinin-3, appears to be beneficial for endurance performance. The lack of α-actinin-3 induces changes in fast-twitch muscle fibers, causing them to adopt characteristics of slow-twitch fibers, such as increased oxidative capacity to generate energy and resistance to fatigue [113,114]. This shift in muscle fiber characteristics may increase aerobic capacity and thıs, beneficits endurance-type activities.

Although several studies have associated the *ACTN3* R577X polymorphism with endurance athletes [114,115], some have reported conflicting results. Recently, Akazawa et al. [43] examined 906 Japanese elite athletes (sprint/power sports, endurance sports, artistic sports, martial arts sports, and ball game sports) and 649 controls. When compared with previous literature, the R allele showed consistent results regarding links to sprint and power sports, while no association was found regarding the X allele between endurance athletes (*n* = 123) and controls (*n* = 718). Similarly, de Albuquerque-Neto et al. [42] found that in Brazilian swimmers (*n* = 123), individuals with the XX genotype were more likely (OR = 1.79) to belong to the athlete group, but there were no significant differences in genotype distribution between athlete and control groups.

#### 3.3.2. β2-Adrenergic Receptor Gene (*ADRB2*)

Another gene of interest is *ADRB2*, which encodes the β_2_-adrenergic receptor. This receptor plays multiple roles in endurance performance, including regulation of cardiovascular function and regulating lipid metabolism, both of which influence body weight and energy availability—critical factors for any endurance athlete [116]. *ADRB2* has two distinct polymorphisms that are thought to affect endurance performance: *ADRB2* Arg16Gly (rs1801253) and *ADRB2* Gln27Glu (rs1042714) [117,118]. The Arg16 allele of the *ADRB2* Arg16Gly (rs1801253) has been theorized to positively affect cardiovascular performance, when compared with the Gyl16 allele. The Gly16 allele, for example, exhibits greater prevalence among sedentary controls [118]. On the other hand, the Glu27 allele of the *ADRB2* Gln27Glu polymorphism has been associated with sprint/power athlete status, as carriers of the Glu27 and Gly16 alleles were more likely to be strength-oriented athletes [117]. Moreover, in patients with heart disease, Gly16 has been associated with a reduced response to exercise training, suggesting that the Gly16Glu27 haplotype might be negatively correlated with endurance status [119]. However, interactions between these polymorphisms and training or environmental factors are not fully understood, and further research is needed to clarify their role in endurance performance. It is possible that the effects of *ADRB2* polymorphisms are modulated by training status, ethnicity, and/or other genetic variants.

#### 3.3.3. Bradykinin β2 Receptor Gene (*BDKRB2*)

Bradykinin is involved in many biological processes, including vasodilation—an important mechanism during endurance exercise. This vasodilation effect occurs when bradykinin binds to its receptor—the bradykinin β2 receptor (B2R) [120]. A polymorphism involving the absence (−9), rather than the presence (+9), of a 9-base pair repeat in exon 1 of *BDKRB2*, the gene that codes B2R, is associated with higher gene transcriptional activity and increased receptor mRNA expression. The −9 allele may benefit endurance performance through several mechanisms. One example being a vasodilator effect, due to an overabundance of BR2 receptors [39,120]. However, evidence regarding the association of *BDKRB2* (−9/+9) with endurance performance and athlete status is mixed. While some studies suggest a positive association, others find no significant effects.

In addition to its role in vasodilation, *BDKRB2* (−9/+9) is thought to be associated with greater muscle contraction efficiency. Saunders et al. [121] found significant differences in male Caucasian triathletes (*n* = 443) competing in the 2000 and 2001 South African Ironman Triathlons, when compared with Caucasian controls (*n* = 203). The −9/−9 genotype of the *BDKRB2* gene was overrepresented in the athlete group, and further analysis showed that this genotype was overrepresented in the fastest finishers. This finding supported the author’s hypothesis that faster finishers have superior endurance versus slower finishers. A study by Sawczuk et al. [119], who attempted to replicate the findings of Saunders et al. [121], reported contrasting data. In their study of 302 Polish elite endurance athletes and 684 controls, no association was found between *BDKRB2* (−9/+9) and endurance performance. A subsequent replication study in 822 Russian elite athletes and 507 controls also failed to find an association [122]. It is important to notethat, in both cited studies the athletes were divided into subgroups based on the anaerobic and aerobic energy system contributions. Nevertheless, the *BDKRB2* gene remains a key factor in cardiovascular physiology, and further research is warranted to elucidate its role in endurance performance [120,123,124].

#### 3.3.4. Peroxisome Proliferator-Activated Receptor Alpha Gene (*PPARA*)

Peroxisome proliferator-activated receptor α (PPARα) is a receptor protein that functions as a transcription factor and plays a regulatory role in energy metabolism, such as controlling glucose and fatty acid uptake [125,126]. *PPAR*α regulates oxidative enzymes such as acyl-CoA oxidase, thereby influencing the type of fuel (e.g., glucose or fatty acid) used during exercise and cardiac function. Substrate regulation during cardiac growth is crucial for cardiac health and longevity [127,128].

The *PPAR*A gene encodes the PPARα receptor and contains a common polymorphism in intron 7, G/C (rs4253778). While both alleles have been associated with athletic performance, the G allele appears to be of greater relevance to endurance performance [129]. This allele is thought to influence the expression of *PPARA*, with changes in circulating PPARα levels affecting the transcriptional activation of *PPARA* target genes [126]. Alsothe G allele has been linked to an increase in type I muscle fibers. In addition, *PPARA* is expressed at higher levels in the type I muscle fibers than in the type II muscle fibers, and the G allele has been associated with overexpression of *PPARA* [127]. This overexpression of *PPARA* may increase fatty acid uptake, potentially benefiting endurance sports by mobilizing the energy stores for prolonged activity. However, the interaction of environmental factors (e.g., sports training, dietary intake) with *PPARA* polymorphisms remains poorly understood, and further studies are needed to explore these relationships. Not all data on *PPARA* G/C are consistent. A study by Eynon et al. [130], examined *PPARA* G/C and *PPARGC1A* Gly482Ser in elite Israeli endurance (*n* = 81) and sprint/power (*n* = 74) athletes, finding no significant differences between these athletic groups and controls (*n* = 240). However, due to the small sample size, the possibility of reporting false negatives does increase, especially with further allocation of the athlete population into smaller subgroups, with almost half being sprint/power athletes.

#### 3.3.5. Uncoupling Protein 2 (*UCP2*) Gene and Uncoupling Protein 3 Gene (*UCP3*)

Uncoupling protein 2 (UCP2) and uncoupling protein 3 (UCP3) are mitochondrial electron transporters involved in ATP production and energy metabolism. *UCP2* is expressed in many tissues, including the kidney, heart, and skeletal muscle, while *UCP3* is mainly expressed in skeletal muscle [131]. Both proteins (*UCP2* and *UCP3)* are thought to play key roles in energy production and regulation of mitochondrial energy metabolism. Moreover, they play a protective role against oxidative damage by influencing reactive oxygen species [131]. The *UCP2* and *UPC3* genes code for uncoupling protein 2 and uncoupling protein 3, respectively. The Ala55Val (rs660339 C/T) polymorphism of *UPC2* has been associated with changes in energy consumption. The T allele was associated with higher VO_2max_ and running success, and was overrepresented in Russian elite athletes [9,132]. Similarly, the T allele of the *UCP3* -55C/T polymorphism (rs1800849) was found to be overrepresented in the same cohort of Russian elite endurance athletes (*n* = 287), highlighting a possible connection.

These findings were however limited to specific populations, and it is unclear whether these associations hold across ethnic groups. In contrast to previous findings, an earlier study by Hudson et al. [133], examined *UCP3* –55C/T and found no association between *UCP3* –55C/T and endurance performance. In this work, 178 triathletes who finished either the 2000 or 2001 marathon were grouped into the fastest (*n* = 89) and slowest (*n* = 89) finishers for genotyping along with 92 healthy Caucasian males as controls. No significant group differences were found regarding the –55C/T genotype. While the evidence is mixed, more recent studies suggest a potential role for UCPs in sports performance. Further research is needed to determine the generalizability of these findings.

#### 3.3.6. Vascular Endothelial Growth Factor-A Gene (*VEGFA*)

Another gene of interest is the Vascular Endothelial Growth Factor-A (*VEGFA*), which plays a significant role in angiogenesis and skeletal muscle adaptations. Angiogenesis, the process of new blood vessel formation, is crucial in endurance exercise because an increase in capillaries within the muscle directly improves gas exchange and thus increases VO_2max_ [134]. *VEGFA*, a glycoprotein encoded by the *VEGFA* gene, regulates angiogenesis and is expressed in skeletal muscle and many other human tissues. A polymorphism in the promoter region of the *VEGFA* gene, G634C (rs2010693), has been shown to influence *VEGFA* gene expression, thereby impacting VEGF protein levels [135]. The C allele of the *VEGFA* G634C has been associated with endurance athlete status [9] and higher VO_2max_ values after 24 weeks of aerobic exercise training [121]. Moreover, the C allele of G634C has been associated with higher VO_2max_ [9,135]. Although not directly contradicting previous findings, the study by Boidin et al. [136] demonstrated that the G allele was associated with better strength adaptations, as compared with VO_2peak_ improvements, in 28 untrained men after 12 sessions of resistance training. A randomized, balanced cross-over design was employed, where the subjects engaged in 12 sessions of resistance training or endurance training over 4 weeks. GG homozygotes showed greater strength gains compared with VO_2peak_ improvements, possibly due to lower circulating VEGFA levels, whereas C-allele carriers may have impaired adaptation to resistance training due to a compromised extracellular matrix. Although research investigating *VEGFA* G634C and its effects on athletic performance is still limited, early studies suggest that this gene is a promising area for future investigation.

The cited literature highlights the complexity of genetic influences on skeletal muscle function and endurance performance. The interactions between different genetic polymorphisms and environmental factors, such as training and diet, may contribute to the variability observed in athletic performance. The genes discussed represent a mix of some of the most studied (*ACTN3* and *ACE*) and less understood (*UCP2* and *UCP3*) indicators in sports genetics.

The field of sports genetics is dynamic and constantly evolving, with new studies frequently challenging existing associations between genetic polymorphisms and elite performance [50,56]. While some polymorphisms maintain their status as significant contributors to athletic performance, others are being reconsidered as new evidence emerges [43,46,50,54]. Advances in methodology and a deeper understanding of genetic mechanisms continue to reshape the landscape of sports genetics, providing new perspectives on previous findings and guiding future research directions [51].

From the discussion above, it is clear that some genes and polymorphisms influence VO_2max_ at least to a degree. However, to fully understand why sports genetics is such an important area, and why more research should be undertaken, it is necessary to discuss the extent to which genetics impact athletic preparation for, and potential in, sport. Understanding these relationships requires extensive research, with diverse and adequately sized populations, to clarify the genetic contributions to endurance performance and adaptation. By exploring the extent to which genetics contribute to athletic potential, we can better understand the importance of genetic factors relative to environmental influences, which is essential for advancing both scientific knowledge and practical applications in athletic training and talent identification.

## 4. Heritability of CRF

Individual variance in VO_2max_, a key determinant of endurance performance, is influenced by both genetic and environmental factors [30,137]. While this review focuses on the genetic contribution to VO_2max,_ it is important to recognize the role of environmental factors, which include a wide range of influences such as training methodology, diet, sleep habits, and even the altitude at which an athlete lives or trains [138,139,140]. These environmental factors may interact with genetic predispositions, further complicating our understanding of VO_2max_ variability. Gene–environment interactions can modulate the expression of genetic potential, making it crucial to consider both factors when examining endurance performance [31].

Heritability, defined as the proportion of phenotypic variance that can be attributed to genetic variation, is a key concept in understanding genetic influences on VO_2max_ [141]. To illuminate the heritability of VO_2max,_ requires careful consideration of inter-individual differences in other features (e.g., fitness level, age, sex), that may influence endurance-related phenotypes [7]. Twin and family studies are particularly valuable in this regard, as they help to control for environmental variance by studying individuals with similar genetic backgrounds, and who share comparable environments [30,135,142].

This is crucial as anthropometric factors (e.g., height, body mass) are often coupled with elite athlete performance, and variance in these factors are tied to genetics, according to evidence obtained from twin, sibling, and family studies [142]. As an example, heritability estimates for height, obtained from a predominantly European cohort, indicate that genetic factors explain around 85% of the variation in height. In contrast, when corrected for height, the variance in body mass determined by genetics is around 40%.

Research on monozygotic (MZ) and dizygotic (DZ) twins is central to heritability studies. Monozygotic twins share nearly 100% of their DNA sequences, while dizygotic twins share approximately 50% of their DNA [143]. This genetic similarity allows researchers to isolate and estimate the genetic contribution to VO_2max_ variability with greater accuracy. Twin studies are particularly useful because they can disentangle genetic and environmental influences. However, they are not without limitations. For example, the assumption that MZ and DZ twins share the same environment may not always be true [144].

Exercise testing is a fundamental component of heritability studies, along with the statistical calculations that determine the heritability estimate. Aerobic exercise tests are typically conducted under standardized laboratory conditions, usually on a cycle ergometer or treadmill. Early studies estimated the heritability of VO_2max_ to be as high as 93% [145]. This study was however limited by a small sample size (*n* = 50), potentially introducing statistical bias or error. Subsequent research has led to more conservative estimates. A more robust and widely cited study by Bouchard et al. [146], part of the HERITAGE Family Study, estimated VO_2max_ heritability to be approximately 47%. This study included a much larger sample of 429 individuals (170 parents and 259 offspring) and controlled for body mass, sex, and age differences when examining VO_2max_. The use of sophisticated statistical models, such as maximum likelihood estimation and variance component analysis, provided a more accurate estimate of heritability than previous studies [146]. These findings suggest that genetic factors play a substantial, but not exclusive, role in determining VO_2max_.

Familial aggregation has also been identified as a significant contributor to VO_2max_ variance [30]. Familial aggregation refers to the clustering of certain traits within families, with genetic and environmental factors again playing a moderating role [30,147]. Because family members often share similar environments as well as genetics, disentangling genetic influences from environmental ones can be challenging. Methods such as adoption studies and the inclusion of unrelated individuals raised in the same environment can help address this issue [148]. It is important to account for these factors when assessing the heritability of VO_2max_ [138,149]. Studies have used various methods to control for familial environmental effects, such as statistical modeling or inclusion of environmental covariates, but further refinement is needed to improve the accuracy of heritability estimates in performance.

Research by Schutte et al. [150], addressed some of these challenges by subjecting adolescent twins and siblings (*n* = 479) to a graded maximal exercise test and two submaximal exercise tests. The authors also tested the validity of submaximal exercise tests in estimating the VO_2max_. This work addressed some gaps in the literature, namely a lack of maximal tests and adolescent populations when examining VO_2max_ heritability. The study included four hundred seventy-nine subjects, consisting of one hundred and twelve MZ pairs and one hundred and nine DZ pairs, as well as thirty-three singleton siblings and two non-twin pairs, providing a large sample size that strengthened its findings. The authors employed a genetic structural equation modeling to analyze the data. Specifically, a trivariate Cholesky decomposition model was used to partition phenotypic variance into additive genetic, dominant genetic, shared environmental, and unique environmental components. The key finding was that additive genetic factors explained 60% of the variance in VO_2max_, while a shared environment contributed 13%, and unique environment accounted for 27% [150]. The meta-analysis, which included twin and sibling studies measuring VO_2max_ in children and young adults, yielded a weighted heritability estimate of 59%, with a 95% confidence interval of 52–66%. This study confirmed a substantial genetic contribution to VO_2max_ variability in adolescents. The phenotypic correlations between measured and predicted VO_2max_, from both submaximal treadmill and cycle ergometer tests, were moderate in strength, due to the poor agreement between predicted and measured heart rate. Nevertheless, the submaximal exercise tests did yield very comparable estimates of VO_2max_ heritability. This suggests that, despite some limitations, submaximal testing may be useful in estimating VO_2max_ for use in heritability studies. The study findings are limited to Caucasian adolescents, although the authors [149] did provide a comprehensive overview of heritability estimates of VO_2max_ among adolescent cohorts.

Miyamoto-Mikami et al. [151], provided corroborating evidence, by conducting a systematic review and meta-analysis of fifteen studies. One of the main differences between Miyamoto-Mikami et al. [150] and Schuttle et al. [150] was the inclusion of a systematic review in the former. Of the fifteen articles reviewed, nine studies were twin studies, and four were family studies, while the remaining two had both family and twin participants. Diversity in sample size was a highlight (*n* = 32 to *n* = 2116), along with all studies (*n* = 15) reporting VO_2max_ or VO_2peak_. The meta-analysis reported that the heritability of VO_2max_, adjusted for body weight and fat-free mass, was 56% and 44%, respectively. The unadjusted heritability was 68%. The heritability estimates showed some heterogeneity; however, this heterogeneity disappeared when the sex of the participants was taken into account. Miyamoto-Mikami et al. [139] also highlighted the need for female-only studies, citing the possibility that the heritability of VO_2max_ might be affected by sex. Lastly, one of the main limitations of the study was that, out of the fifteen articles examined, only three articles provided a 95% confidence interval regarding heritability estimates.

Given the above, the genetic component of VO_2max_ has been estimated to account for 44% to 68% of the variance in performance [30,146,150,151]. These consistent findings across populations and methodologies underscore the important role of genetics in determining VO_2max_. However, the degree of unexplained variance highlights the importance of non-genetic factors (e.g., environment, motivation, opportunities) or complex epigenetic (i.e gene–environment) interplay. The importance of environmental factors, such as training intensity, nutritional status, and lifestyle habits, have been highlighted [152]. Gene–environment interactions, in which environmental factors can influence the expression of genetic potential, further complicate the understanding of VO_2max_ variability [153]. In this regard, future research should focus on identifying the specific genes involved and how they interact with environmental factors to influence VO_2max_ using approaches such as genome-wide association studies (GWAS) and epigenetic analyses [31].

## 5. Genetic and Epigenetic Factors Influencing VO_2max_ Trainability

Another important genetic factor that contributes to VO_2max_ variation is the individual response of the cardiorespiratory system to endurance training. While the physiological mechanisms underlying adaptations to endurance training are well documented [7,16,23,24,28,139], there is evidence of notable interindividual differences in how the cardiorespiratory system adapts to such training [29]. These differences, often referred to as “trainability”, indicate that not all individuals experience the same improvements in VO_2max_ in response to identical training stimuli [154]. However, there is scarce discussion regarding the specific genetic factors that contribute to these interindividual differences. Understanding the genetic basis of trainability is crucial for optimizing training programs and maximizing athletic performance.

A pivotal study by Bouchard et al. [29] examined the trainability of maximal oxygen uptake after a 20-week endurance training (ET) program in 483 sedentary individuals from 99 nuclear families (parents and their offspring). Participants underwent treadmill-based ET three times per week, with intensity increasing from 55% to 75% of baseline heart rate and session duration up to 50 min. Improvements in VO_2max_ varied considerably among participants, ranging from 0.1 L min^−1^ to 0.7 L min^−1^. The heritability of training response was estimated at 47%, with between-families variation being 2.5 times greater than within-families variation [30]. This suggests that genetic factors significantly influence how individuals respond to endurance training. A maternal heritability component of 28% also emerged, suggesting a potential mitochondrial influence on genetic variation in training response. It is noteworthy that some individuals showed little to no improvement in VO_2max_, while others showing significant gains. This variability may be due to specific genetic variants that can enhance or possibly limit adaptability to exercise.

In agreement with the cited work, Timmons [154], found that even when individuals adhere to identical standardized training protocols, there are still significant differences in VO_2max_ improvements. This variation in “trainability”, suggests the existence of “high responders”, “low responders”, and even “non-responders” to endurance training. This interindividual variability underscores the role of genetic factors in influencing how effectively an individual’s cardiorespiratory fitness improves with training. Timmons discusses how genetic predisposition can account for a significant portion of the variability in training responses. For example, some individuals may possess genetic variants that enhance mitochondrial biogenesis, capillary density, or oxygen utilization efficiency, leading to greater improvements in VO_2max_ [155].

Identifying genetic markers associated with trainability could facilitate personalized exercise prescriptions. That is, by understanding an individual’s genetic profile, coaches and clinicians could tailor exercise programs to maximize effectiveness and minimize the risk of overtraining or injury. This personalized approach would be particularly beneficial for elite athletes who seek to optimize performance at the upper limits of human performance where marginal gains are crucial for success and for those individuals seeking to improve cardiovascular health more generally [154].

Pursuant to the work by Bouchard et al. [30] studies have further delved into the genetic basis of VO_2max_ training adaptations, focusing on submaximal exercise [29,138,149]. These studies found that the training responses were largely independent of age, sex, or body mass, but the relative heritability of the training response was lower, with a small but significant familial component. The same research also highlighted the importance of environmental factors and gene–environment interactions in determining exercise trainability [29]. Another key finding was the need for standardizing testing protocols, especially when analysing specific components of VO_2max_ (e.g., heart rate, systolic blood pressure).

Building on these findings, Bouchard et al. [156] conducted the first GWAS to identify specific genetic variants associated with VO_2max_ trainability. To do this, they examined 324,611 single nucleotide polymorphisms (SNPs) in individuals of Caucasian origin, with a total of 483 adults from 99 families completing the study. After 20 weeks of endurance training, the authors identified 39 SNPs that were associated with an increase in VO_2max._ Further analysis with a multivariate regression model identified 21 SNPs that collectively explained approximately 49% of the variance in VO_2max_ trainability, indicating a significant relationship between these genetic variants and the training response. Notably, six SNPs each accounted for at least 3% of the variance.

The six SNPs showing the strongest association with increased VO_2max_ were rs10499043 (located in the *PRDM1* gene), rs1535628 (*GRIN3A*), rs4973706 (*KCNH8*), rs12115454 (*C9orf27*), rs6552828 (*ACSL1*), and rs11715829 (*ZIC4*). It is notable that these SNPs are involved in various biological processes, including fatty acid metabolism and neuronal function.

To validate their results, the authors attempted to replicate the associations in several cohorts, including subjects of African origin from the HERITAGE study (*n* = 247), the Dose Response to Exercise Training (DREW) Study (*n* = 112), and the Studies of a Targeted Risk Reduction Intervention Through Defined Exercise (STRRIDE) (*n* = 183). This work produced mixed results, reflecting the complexity of genetic influences on VO_2max_ trainability and, at the same time, the inherent challenges of replicating associations across different populations and different training protocols.

While the initial cohort size (*n* = 483) in the HERITAGE Family Study is substantial for an exercise intervention study, it may still limit the power to detect associations and replicate findings, as compared to a larger GWAS that often include thousands of participants. Furthermore, differences in exercise modalities and training intensities between studies is a delimiting factor that may explain discrepant results.

Nonetheless, the finding that approximately 49% of the variance in VO_2max_ trainability could be predicted by 21 SNPs underscores the substantial genetic component involved. However, limitations of such studies should be acknowledged, including challenges associated with sample sizes in exercise genomics research, which may have contributed to difficulties in detecting smaller genetic effects and replicating findings. In addition, analyzing heritability for individual physiological components of VO_2max_, such as heart rate or blood pressure, inherently reduces the heritability score because VO_2max_ is a polygenic trait influenced by multiple genes acting on different physiological pathways. Finally, the replication of genetic associations across diverse populations remains challenging, due to individual differences in genetic background and environmental factors.

In a meta-analysis by Montero and Lundby [157], the extent of non-responsiveness to endurance training was questioned. The suggestion was that inadequate training stimuli, rather than genetic factors alone, may explain the lack of improvement in some individuals. By increasing the training dose—through higher intensity, duration, or frequency—all participants showed significant improvements in VO_2max_. This finding challenges the notion of “non-responders” and highlights the importance of a sufficient training stimulus. The authors argued that individualized training programs that take into account an individual’s initial fitness level and adjust the training load accordingly can lead to positive adaptations for all. This perspective suggests that there is a highly individualized training threshold, which may itself be influenced by genetic factors. Exceeding this threshold is necessary to induce improvements in VO_2max_. Therefore, optimization of training protocols is essential before concluding that genetic factors are the primary reason for lack of response to endurance training.

Bacon et al. [158] supported the need for standardization of training protocols in their meta-analysis, noting that individuals who trained more frequently and at a higher percentage of VO_2max_ (up to 90%, rather than 50% or 60%) showed greater adaptations. The authors also highlighted the effectiveness of interval training protocols. The individuals tested consistently displayed either no response, or a hyper-response to endurance training. This persistency in individual variability, regardless of training intensity or protocol, suggests that genetic factors may play a significant role in determining individual responsiveness to different training stimuli.

Despite the insights provided by these studies, it is important to note that many lacked a genetic analysis component, leaving the genetic basis for the observed heritability largely unexplored. A literature review suggests that the heritability of the training response for VO_2max_ ranges from approximately 50% to 60% [20,135,150,153,159]. These findings reinforce the importance of genetic factors in determining individual variability in response to endurance training and highlight the need for further research into the specific genes involved.

Advancements in genomic technologies, such as whole-genome sequencing and epigenetic profiling, offer potential for gaining deeper insights into the genetic determinants of VO_2max_ trainability [12]. Epigenetics refers to heritable changes in gene expression that do not alter the underlying DNA sequence. Studies have begun to explore the significant role of epigenetic modifications—including DNA methylation, histone modifications, and non-coding RNAs—in influencing VO_2max_ responses to exercise training [160,161].

DNA methylation, the addition of a methyl group to cytosine residues in DNA, can modulate gene expression by altering the accessibility of the transcriptional machinery to the DNA strand. Exercise-induced changes in DNA methylation have been observed in genes related to mitochondrial biogenesis and energy metabolism, such as *PGC-1α* and *PDK4* (Pyruvate Dehydrogenase Kinase 4), both of which have been associated with VO_2max_ improvements following endurance training [162,163]. For example, acute endurance exercise has been shown to decrease methylation of the *PGC-1α* promoter region, leading to increased gene expression and improved mitochondrial function [162].

Histone modifications, including acetylation and methylation, also play a critical role in regulating gene expression by affecting chromatin structure. Endurance training can lead to increased histone acetylation in skeletal muscle, promoting a more open chromatin conformation and facilitating the transcription of genes involved in oxidative metabolism [164]. This epigenetic remodelling enhances the muscle’s capacity for aerobic energy production, contributing to improvements in VO_2max_.

Non-coding RNAs, particularly microRNAs (miRNAs), are short RNA molecules that regulate gene expression post-transcriptionally. Exercise alters the expression profiles of specific miRNAs in skeletal muscle, affecting pathways involved in angiogenesis, muscle fiber type specification, and mitochondrial adaptation [165,166]. For example, miR-1 and miR-133a are modulated by endurance exercise and are involved in muscle differentiation and adaptative processes [166]. These miRNA-mediated regulatory mechanisms may contribute to individual differences in VO_2max_ trainability.

Furthermore, epigenetic modifications can have lasting effects, potentially influencing long-term adaptations to training and even transgenerational inheritance of fitness traits [160]. Integrating epigenetic data with genetic and transcriptomic information could improve our understanding of the molecular mechanisms underlying VO_2max_ adaptations and offers new targets for personalized training interventions [167]. For example, individuals with specific epigenetic markers could be identified as high responders to endurance training, allowing for the optimization of training programs to maximize VO_2max_ [161]. Conversely, recognition of epigenetic profiles associated with lower trainability could prompt a new training approach or inform an early intervention (e.g., dietary modifications), to enhance responsiveness.

In summary, understanding the genetic and epigenetic determinants of VO_2max_ adaptation is critical for coaches, athletes, and clinicians. This knowledge could be used to optimize training programs, by tailoring intensity and volume to individual needs, potentially improving training efficiency and performance outcomes. Future advances in genetic and epigenetic technologies may enable even more precise and effective training prescription at the individual level, ushering in an era of truly personalized exercise prescriptions.

## 6. Challenges, Implications, and Future Directions in Endurance Genetics Research

Understanding genetic influences on endurance performance has broad implications, with significant benefits for both sports and medicine. In sports, knowledge of genetic factors can improve the accuracy and standardization of testing for gene doping [168]. Gene doping refers to the misuse of gene therapy techniques to enhance athletic performance, posing a significant challenge to the World Anti-Doping Agency (WADA) [169]. Medicine is increasingly moving toward personalized gene therapy strategies for many diseases, including cancer and genetic disorders [170,171]. As gene therapy becomes more widespread, particularly with advances such as CRISPR (Clustered Regularly Interspaced Short Palindromic Repeats), concerns about gene doping in sport are likely to increase [170,171]. Although no athlete has been formally accused of gene doping [8], the increasing accessibility of genetic technologies like CRISPR and their potential to improve performance makes this a future concern [169]. A comprehensive understanding of specific genes and their polymorphisms, especially in a sport context, will be crucial for developing effective anti-doping strategies [168], and may minimize false positives in gene-doping tests by established a detailed genetic database for comparative purposes.

Beyond anti-doping effects, understanding an individual’s genetic profile provides valuable information for creating personalized training regimens and injury prevention strategies [172,173]. As an example, certain genes such as *ACE*, *BDKRB2*, and *IL*-6 have been linked to injury mechanisms [39,52]. For exercise scientists and coaches, knowledge of these genetic factors could better inform the development of personalized training programs that incorporate specific conditioning exercises, recovery protocols, and nutritional interventions to optimize performance and reduce injury risk [174,175]. A personalized approach would not only enhance athletic performance but could also support the long-term health and well-being of athletes.

Practical applications of genetic profiling are beginning to emerge in sports settings. For instance, genetic information is being used to tailor nutrition plans based on genes affecting nutrient metabolism, such as caffeine sensitivity or vitamin D absorption [176]. Additionally, understanding genetic predispositions to certain injuries can inform preventive strategies. For example, polymorphisms in the *TNC* (Tenascin C), *COL5A1* (Collagen type V, alpha 1 chain), and *MMP3* (Matrix Metallopeptidase 3) genes co-segregate with chronic Achilles tendinopathy, suggesting that athletes with these variants may benefit from specific training modifications [177]. Furthermore, the *ACTN3* R577X polymorphism has been associated with injury incidence and severity in professional football players, suggesting that genetic profiling could help identify athletes at higher risk for specific injuries and guide personalized training and prevention programs [178].

Although genetic testing for sports performance and injury susceptibility has been conducted in elite UK sports, recent surveys indicate that its usage remains limited [179,180]. Only a small proportion of athletes (≤17%) and support staff (≤8%) reported engagement with genetic testing. However, there is a strong belief among athletes and support staff (≥79%) that genetics plays a powerful role in determining elite status, with many expressing a willingness to incorporate genetic testing to personalize training and minimize injury risk. Therefore, although opinions vary with regards to using genetic data for talent identification or influencing athlete selection and employment status [179], the vast majorit of elite athletes and coaches (in the UK) show broad acceptance and application of genetic evidence.

Recent polygenic risk models have highlighted the importance of the *VEGFA* gene in susceptibility to musculoskeletal injury, particularly anterior cruciate ligament (ACL) rupture. One study [181] identified the *VEGFA* rs699947 CC and rs2010963 GC genotypes as significant predictors of ACL rupture risk. These findings, along with factors such as age and BMI, emphasize the importance of genes associated with the angiogenesis pathway in injury risk, and highlight the potential for incorporating such genetic insights into prevention and rehabilitation strategies.

Despite significant progress, research indicates that the majority of genetic studies on injury susceptibility are underpowered, limiting their ability to detect small effect sizes [182]. More than 70 loci have been implicated in various soft tissue injuries, including genes encoding both collagenous and non-collagenous matrix proteins. Large-scale data and collaborative consortia are needed to effectively pool resources and validate findings, thereby ensuring the identification of genetic targets with greater clinical significance. Many direct-to-consumer genetic tests marketed to athletes are premature [183], as they are often not interpreted alongside clinical indicators or lifestyle factors by qualified professionals. Current practice should focus on known risk factors, such as family history and previous injuries, to guide prevention strategies.

The use of genetic profiling in athletes comes with its own set of challenges. While commercially available genetic tests are sometimes used for talent identification or genetic profiling, the information provided is based on limited data and sometimes equivocal evidence [184,185]. As such, these tests should not be used as the sole basis for talent identification. Furthermore, genetic testing in this manner raises ethical questions related to privacy, consent, and potential discrimination [186], and they are prone to error and bias, which can affect the validity of results [184,185]. A critical review by Collins and September [187] found that current evidence does not support the use of common polymorphisms (e.g., *COL1A1* rs1800012, *COL5A1* rs12722, *GDF5* rs143383) in commercial genetic tests for injury risk, due to low sensitivity and lack of validation. To summarize, whilst genetic profiling shows some promise, its application in assessing injury susceptibility and subsequent exercise programming, requires further rigorous investigation.

Our review highlights several inconsistencies and contradictions in the literature regarding the association between specific genetic polymorphisms and endurance performance. Polymorphisms such as *ACE* I/D and *ACTN3* R577X, despite extensive study, have yielded mixed results across different populations and sports disciplines. These inconsistencies may be due to factors such as small sample sizes, population stratification, differences in athlete classification, and variability in environmental factors such as training and diet. The lack of standardized methodologies and potential publication bias toward positive findings further complicate the interpretation of these studies. Such biases may overemphasize the importance of certain genetic markers while underreporting negative or inconclusive results.

These inconsistencies have important implications. They suggest that the relationship between genetics and endurance performance is highly complex and influenced by multiple interacting factors. Relying on single genetic markers to predict performance or identify talent may be unreliable and potentially misleading. This underscores the need for a more holistic approach that considers the polygenic nature of endurance traits and the significant role of environmental influences.

In addition to physiological factors, emerging evidence highlights the importance of psychogenetics—the study of how genetic polymorphisms affect psychological traits relevant to athletic performance. Traits such as motivation, resilience, and stress response are essential for training adherence and performance in endurance sports. Genetic polymorphisms, particularly those in the dopaminergic system, can significantly impact these traits. Our recent review [188] discusses how genetic and epigenetic factors influence dopamine pathways, thereby influencing personality traits and reward sensitivity. This psychogenetic perspective is essential for understanding the holistic nature of endurance performance, as psychological resilience and motivation may be as critical as physical capacity. Incorporating these findings into training strategies could lead to more comprehensive and personalized athlete development.

The field of epigenetics adds another layer of complexity. Epigenetic modifications resulting from environmental factors such as training load, diet, and lifestyle can influence gene expression and athletic performance [167]. These modifications may be sport-specific and occur at different times and rates. For example, a mixed martial arts (MMA) fighter may experience epigenetic changes related to weight cutting, whereas a basketball player may experience different changes due to the demands of a long competitive season. Therefore, genetic tests that do not account for epigenetic or environmental factors may provide an incomplete picture. Global standardization of epigenetic or genetic tastings should be established before any genetic profiling tests are included in any sports programs.

When it comes to large-scale genetics tests, the possibility of uncovering genetic predispositions to disease is always a concern. The inadvertent discovery of a genetic disease risk might have unintended consequences and raises many ethical concerns, such as the psychological impact on the athlete and the potential for unnecessary medical interventions [184]. In addition, the exclusion of an individual from a sport on the basis of a genetic test—which may produce false positives or negatives—should be carefully considered by governing sports bodies.

Despite these challenges, the integration of genetic knowledge into traditional training methods holds great promise. With a cautious and evidence-based approach, coaches and practitioners can use genetic information as one of many tools to enhance athlete development. For example, genetic testing can complement physiological assessments to provide a more holistic understanding of an athlete’s abilities and needs.

To advance the field and address current gaps, future research should aim to improve study designs and methodologies. One critical improvement is the standardization of athlete classification and phenotyping protocols. This includes clear definitions of what constitutes an elite endurance athlete, standardized testing procedures for performance metrics such as VO_2max_, and detailed recording of environmental factors such as training regimens and dietary habits. Such standardization would reduce variability and improve the comparability of studies.

Additionally, increasing sample sizes through international collaborations and the creation of large consortia can enhance statistical power and the ability to detect true genetic associations. Inclusion of diverse populations is essential to account for population stratification and to ensure that findings are generalizable across different ethnic groups. Longitudinal studies that track athletes over time can provide insights into how genetic factors influence performance development and how gene–environment interactions play out over the course of an athletic career.

For future research, it is essential to adopt polygenic approaches that incorporate physiological analyses to explore biological mechanisms underlying genetic associations. By focusing on multiple genes and their interactions, researchers can gain a more comprehensive understanding of the complex factors influencing endurance performance. GWAS and polygenic risk scores can help identify the cumulative effects of multiple genetic variants [189]. Studies should also consider environmental and epigenetic factors alongside traditional genetic approaches to provide a more comprehensive understanding of endurance performance. This integrated perspective recognizes that genetics is only one piece of the puzzle and that lifestyle and environmental factors also play an important role. Integrative omics approaches that combine genomics, transcriptomics, proteomics, and metabolomics can provide deeper insights into the molecular pathways involved in endurance adaptations [190].

In addition, sport-specific research should be conducted with large and diverse sample sizes to better understand how polymorphisms might affect performance in different types of sports and populations [191]. Key polymorphisms, such as *ACE* I/D, *BDKRB2* −9/+9, *PPARGC1A* Gly482Ser, and *PPARA* G/C, should continue to be a focus of research, especially when examined alongside newer polymorphisms that may be understudied or lack extensive evidence. Genes like *VEGF* and *UCP2-3* and their polymorphisms should be subjected to replication studies, and more studies on *GNB3* and *CD36* are required.

Investigating these polymorphisms in the context of specific physiological systems and their effects on endurance performance could provide valuable insights for both sports science and medicine, potentially leading to improved training methods and therapeutic interventions.

Moreover, advanced statistical methods and technologies should be used to deal with the complexity of genetic data. Machine learning algorithms, network analysis, and other computational approaches can identify patterns and interactions among multiple genetic polymorphisms that traditional statistical methods might overlook. These methods can also account for gene–gene and gene–environment interactions, providing a more nuanced understanding of the genetic architecture of endurance performance.

Despite these promising applications, advancing our understanding of the genetic determinants of endurance performance requires overcoming several challenges. Heritability studies have provided crucial insights into the broader role of genetics in endurance performance. However, these studies often lack the depth required to identify specific genes and their associations with performance-related traits. To address this limitation, genetic association studies have become the most common approach in sports genetics, providing the necessary depth to explore specific genetic influences on athletic performance [36]. Advances in genotyping technologies and statistical methods have facilitated this shift, allowing more precise identification of genetic factors contributing to endurance. However, genetic association studies, including GWAS, often explain only a small fraction of heritability, a phenomenon known as “missing heritability” [192]. Integrating epigenetic analyses and examining gene–environment interactions are critical to capturing the full spectrum of genetic contributions to endurance performance [167].

Another critical area of study involves gene–gene or SNP–SNP interactions, particularly when analyzing polymorphisms from a physiological perspective in sports. Athletes typically carry multiple polymorphisms related to athletic performance, which can influence molecular pathways in different ways, resulting in either additive or detrimental effects on endurance. Several studies have explored the combined influence of multiple polymorphisms on endurance performance. For example, Ruiz et al. [193] investigated the interaction between *ACE* I/D and *ACTN3* R577X polymorphisms, finding that certain genotype combinations were more prevalent in elite endurance athletes compared with controls. Another study by [191] further demonstrated the polygenic nature of endurance by evaluating a panel of polymorphisms and their collective effect on athlete status. Our recent study [194] also investigated the genetic profiles of sport climbers of Japanese, Polish, and Russian descent, analyzing the *ACTN3* R577X, *ACE* I/D, *CKM* (rs8111989), and *TRHR* (rs7832552) polymorphisms in 258 climbers and 1151 controls. While no significant differences were found within ethnic groups, a meta-analysis revealed a higher frequency of the *ACTN3* X allele in climbers compared with controls, underscoring the potential association of the allele with climbing status. This finding highlights the importance of incorporating multi-ethnic cohorts and conducting meta-analyses to identify genetic associations that may not be evident in isolated populations. It also emphasizes the significance of considering coexisting genetic polymorphisms when assessing athletic potential.

In addition, product–product interactions, when modulated by SNP–SNP interactions, add another layer of complexity to association studies [195]. This complexity is further increased by epistasis, where the combined effect of multiple genes results in completely different phenotypes [196]. Investigating coexisting polymorphisms requires larger sample sizes to achieve adequate statistical power. The increased number of variables and potential interactions may reduce the ability to detect significant associations if the study is not adequately powered. This requirement underscores the importance of international collaborations and data-sharing initiatives to pool resources and participants.

To mitigate these challenges, future studies should use rigorous study designs that include appropriate control groups, blinding, and statistical adjustments for multiple testing. Researchers should also ensure transparent reporting of both positive and negative findings to reduce publication bias. Standardizing genotyping methods and ensuring quality control measures, such as checking for Hardy–Weinberg equilibrium and genotyping reproducibility, are essential to the validity of genetic association studies.

Several solutions have been proposed to alleviate this increased complexity, such as the implementation of total genotype score (TGS) by [153]. TGS uses generated data of 23 polymorphisms that are closely related to endurance, and the individual is scored according to the optimal composition of desired polymorphisms. While TGS might provide data on the optimal polygenic profile, it is not a one-size-fits-all solution, as due to the polygenic nature of endurance, TGS should not be used as the sole predictor of sports talent nor used as the sole determinant of SNP–SNP interactions, as the evidence is unequivocal [197,198]. Along with TGS and GWAS, advanced statistical methods, such as machine learning algorithms and network analysis, have been employed to handle the complexity of multiple genetic interactions. These approaches can identify patterns and relationships that traditional statistical methods might overlook. For example, machine learning models have been used to predict endurance performance based on a combination of genetic markers [199], demonstrating their potential in sports genetics research.

Polygenic risk scores (PRS) offer a way to quantify the cumulative effect of multiple genetic variants associated with endurance performance. By aggregating the effects of multiple SNPs, PRS can provide an overall genetic predisposition score for an individual athlete. This approach has been applied to other complex traits and diseases and holds promise for sports genetics, although its application requires careful validation [200]. The study of coexisting polymorphisms and their collective effects on endurance performance is critical to advancing our understanding of the genetic basis of athletic ability. Future research should focus on the integration of multiple genetic factors using robust statistical models and larger, more diverse cohorts to capture the complex interplay of genes influencing endurance.

In summary, while the practical application of genetic profiling in athletes is still in its early stages and faces significant challenges, it holds great promise for enhancing personalized training interventions and optimizing performance. Ongoing research and dialogue are needed to fully realize the potential benefits while mitigating risks and ethical concerns.

Even so, genetic studies in sports science involve numerous variables that must be carefully managed. Challenges such as small sample sizes, population stratification, and the complex interaction of genetic and environmental factors can affect study results. Standardizing methods across studies is critical to minimize inconsistencies and increase the reliability of results. It is critical to consider the hierarchy of evidence and address potential limitations and variables that may influence study results.

Addressing these challenges will require a concerted effort by the research community. Establishing international consortia and collaborations can facilitate data sharing and pooling of resources, thereby increasing sample size and diversity. Developing standardized protocols for data collection, genotyping, and statistical analysis can reduce methodological variability and improve study comparability. Encouraging the publication of replication studies and negative findings can help balance the literature and reduce publication bias.

Several strategies can be used to overcome these sample size limitations. First, establishing international collaborations and consortia can pool data from multiple research groups, thereby increasing the total number of participants. This approach not only increases statistical power but also promotes the inclusion of diverse populations, thereby improving the generalizability of findings [201]. Second, using meta-analytic techniques to combine data from existing studies can effectively increase sample sizes. By aggregating the results of multiple small studies, researchers can achieve greater statistical power and more robust conclusions [202]. Third, recruiting sub-elite athletes and well-trained individuals as additional cohorts can expand the participant pool. Although they may not be elite athletes, their inclusion can provide valuable insights into genetic factors influencing endurance performance [203]. Fourth, integrating data from biobanks and large-scale population studies that include performance-related phenotypes can complement athlete cohorts. This approach leverages existing datasets to identify genetic associations relevant to endurance without the need for new, large-scale athlete recruitment [204]. Fifth, applying advanced statistical methods, such as machine learning algorithms and polygenic risk scoring, can maximize the information gained from smaller samples by identifying complex genetic patterns associated with endurance performance [205]. Finally, promoting open data sharing and establishing centralized databases of athlete genetic information can facilitate larger combined analyses and replication studies. By adopting these strategies, researchers can mitigate the challenges posed by small sample sizes, thereby increasing the reliability and validity of genetic association findings.

Through rigorous study designs, increased collaboration, open data sharing, and methodological consistency, the field can further advance our understanding of the genetic determinants of elite endurance performance [185]. This progress will not only enhance the scientific community’s knowledge but also support the practical applications in sports and medicine discussed earlier, ultimately leading to improved athletic performance, better health outcomes, and more effective therapeutic strategies.

In summary, understanding the genetic and epigenetic factors influencing endurance performance has significant implications for sports and medicine. While challenges remain, advances in genetic and genomic technologies, coupled with collaborative research efforts, hold promise for uncovering the complex biological underpinnings of endurance. This knowledge can lead to personalized training strategies, injury prevention, and improved health outcomes, ultimately enhancing athletic performance and contributing to medical advancements.

## 7. Conclusions

Elite endurance athletes represent a small but important group in the study of sports genetics. These athletes are typically distinguished by their participation at the highest levels of competition, (e.g., Olympic Games) and are characterized by a high maximal oxygen uptake (VO_2max_), a key determinant of endurance performance.

Genetics plays a key role in determining elite athlete status, particularly in relation to VO_2max_. Studies of the heritability of VO_2max_ and its response to training suggest that genetics accounts for 44% to 68% of the variation in this trait among athletes, with adjusted heritability estimates of 44% to 56% when controlling for factors like body weight and fat-free mass. As a polygenic trait, VO_2max_ is influenced by multiple genes and polymorphisms, each of which affects different physiological systems, such as the cardiovascular system, the musculoskeletal system, and the respiratory system. Numerous polymorphisms have been associated with endurance performance and the status of elite endurance athletes, some of which have been highlighted in this review. Understanding the methodologies used in genetic studies is important when investigating these polymorphisms. The most common types of studies in sports genetics are association studies, which include genetic association studies, case-control studies, linkage analysis studies, and genome-wide association studies (GWAS). Each type of study has its strengths and limitations, but a common challenge across all types is the issue of small sample sizes. Studies with an inadequate sample size are more prone to false-positive errors, which can compromise the validity and replicability of their findings. Additionally, researchers should focus on studying more homogeneous groups to reduce variability while also including diverse populations to capture the full spectrum of genetic variability and improve the generalizability of genetic associations.

The literature search conducted for this narrative review aimed to identify key findings and gaps in current research on the genetic determinants of endurance performance. While novel findings have emerged, some results, such as those related to the *ACE* I/D polymorphism, remain inconclusive. This highlights the need for further research with larger sample sizes. By studying both homogeneous and diverse groups, scientists can control for confounding variables and better understand the specific genetic factors influencing endurance performance. Future research should be based on a deep physiological understanding and take a holistic approach by examining gene products and their interactions within and between systems in the human body. Moreover, incorporating multi-omics approaches and considering environmental and epigenetic factors will enhance our understanding of how genetics interacts with training and lifestyle to influence endurance performance.

Despite the challenges, the integration of genetics with epigenetics and other “omics” approaches offers a promising avenue for future research. This holistic perspective has the potential to reveal the complex interactions between genes, environment, and training, and to improve our understanding of the biological mechanisms underlying endurance performance.

In conclusion, while significant progress has been made in understanding the genetic determinants of elite endurance athlete status and performance, this narrative review contributes by highlighting recent shifts in research focus, identifying methodological challenges, and suggesting specific strategies for future studies. Continued research with robust methodologies and larger sample sizes, as well as studies focusing on standardized classification of sports and more homogeneous groups, will be essential to unravel the complex genetic architecture underlying endurance performance. As our understanding of these genetic determinants continues to evolve, sports genetics is likely to play a pivotal role in enhancing athletic performance, personalizing training programs, and identifying potential health risks associated with high-intensity sports. The potential for new discoveries is vast, and continued research in this area is essential to fully realize the benefits of genetic insights in sports science.

## Figures and Tables

**Table 1:** Study quality scoring assessment system.

Item	Criteria
1. Control group	Was the control/comparison group equal to or larger in size than the case group, and was it described in such a way that it could be replicated or stated or inferred that the ethnicity of the control group was not different from that of the case group? Note: If the control group from a previous study was used and referenced, the referenced study was retrieved and the control group analyzed as above. No score was assigned to cohort studies for this item.
2. Hardy–Weinberg equilibrium	Were the groups included in the study assessed to determine whether they were in Hardy–Weinberg equilibrium?
3. Case group/whole group	Is the definition of the case group adequate to allow replication? For cohort studies, is the description of the whole group sufficient for replication?
4. Primer	Were the primer sequences provided, or was a reference to them given?
5. Reproducibility of genotyping	Was the description of genotyping methods sufficient to allow replication, or was a reference providing this information given? And was the validity of the genotyping technique checked by performing a second assay technique, by validating the accuracy of the assay used, or was a reference to a validation study given?
6. Blinding	Were genotyping staff blinded to group allocation or phenotypic data?
7. Power calculation	Was a power calculation performed either prospectively or retrospectively?
8. Statistics	Were major findings presented with well-described tests of significance, including *p*-values, odds ratios, or confidence intervals?
9. Corrected statistics	When more than one genetic polymorphism was studied, were statistical corrections made to account for the increased risk of a false positive? Note: Studies using one-tailed significance testing were also scored as zero. Those testing one genetic polymorphism were scored as one.
10. Independent replication	Was a secondary confirmatory study performed, or does the study specifically state it is being performed to confirm results from an earlier study?

Each item was scored ‘1’ if the criterion was met (‘Yes’) and ‘0’ if not (‘No’). The scores were then summed to give a final score ranging from 0 to 10 for case–control studies and 0 to 9 for cohort studies.

## Data Availability

No new data were created or analyzed in this study.
